# Curcumin inhibits ferroptosis-mediated vascular occlusion by regulating the CXCL10/CXCR3 axis in retinopathy of prematurity

**DOI:** 10.1186/s10020-025-01161-1

**Published:** 2025-03-24

**Authors:** Rui Niu, Jing Wang, Xiaolin Pan, Min Ran, Peng Hao, Wei Zhang, Yatu Guo, Wei Zhang

**Affiliations:** 1 Clinical College of Ophthalmology, Tianjin Medical University, Tianjin, China; 2 Tianjin Key Laboratory of Ophthalmology and Visual Science, Tianjin Eye Institute, Tianjin Eye Hospital, Tianjin, China; 3 Hebei Eye Hospital, Xingtai, China; 4 School of Medicine, Nankai University, Tianjin, China; 5 Ophthalmology, Minda Hospital of Hubei Minzu University, Enshi, China

**Keywords:** CXCL10, CXCR3, Curcumin, Ferroptosis, ROP

## Abstract

Retinopathy of prematurity (ROP) is a disorder that causes blindness in children at a high incidence. Retinal endothelial cells are damaged by variations in oxygen partial pressure, which leads to vascular obstruction and, eventually, ischemia and hypoxia, which cause the formation of new blood vessels. However, little is known about the molecular mechanism of hyperoxic vascular occlusion. High oxygen levels are thought to cause ferroptosis. In this study, experiments with both animal and in vitro models demonstrated that elevated expression of C-X-C motif chemokine ligand 10 (CXCL10)/C-X-C motif chemokine receptor 3 (CXCR3) in retinal vascular endothelial cells induced ferroptosis. Curcumin decreased ferroptosis by inhibiting the production of CXCL10/CXCR3. Curcumin also preserved distal sprouts and filopodia, increasing tip cell and astrocyte counts. As a result, we hypothesize that curcumin reduces ferroptosis and preserves retinal blood vessels under hyperoxic conditions by suppressing the CXCL10/CXCR3 axis. Coimmunoprecipitation (COIP) data were used to determine which proteins interact with CXCR3 during ferroptosis. For the first time, our study applied curcumin to treat eye diseases in oxygen-induced retinopathy (OIR) mice and explored the underlying mechanism in cell experiments, laying the foundation for clinical patients to use this drug. Exploring the interaction between CXCL10/CXCR3 and ferroptosis provides an experimental basis for using the CXCL10/CXCR3 axis as a therapeutic target for the treatment of ROP ophthalmopathy.

**Graphical Abstract:**

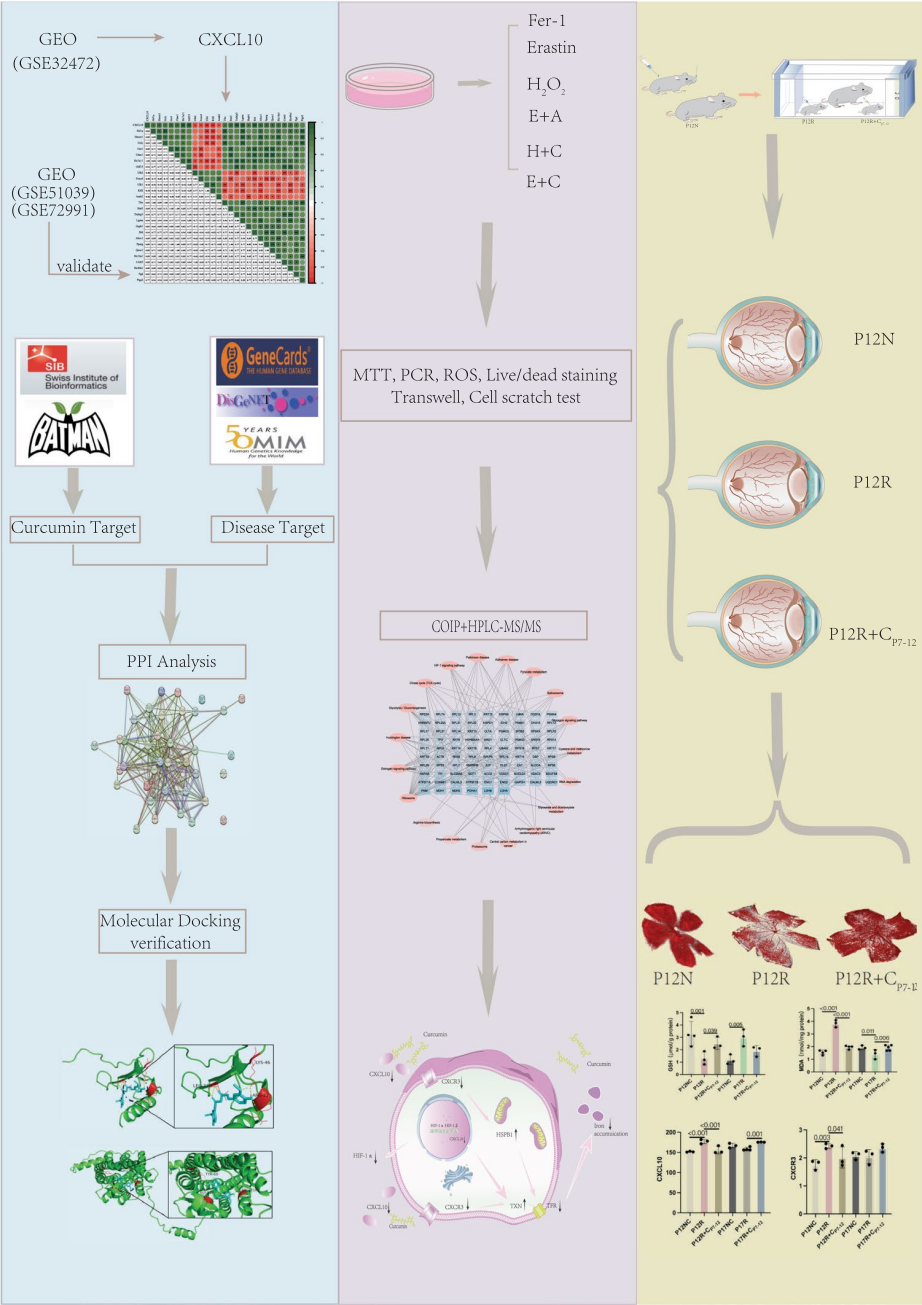

**Supplementary Information:**

The online version contains supplementary material available at 10.1186/s10020-025-01161-1.

## Introduction

One of the major causes of blindness and visual impairment in children worldwide is ROP (Sabri et al. 2022). With the increase in the survival rate of premature infants, the incidence of this disease has increased (Sabri et al. 2022). ROP and its development are mostly linked to high oxygen exposure and variations in oxygen levels before the retina is fully matured. ROP is divided into two stages: hyperoxia and hypoxia. During the hyperoxia phase of ROP, retinal microvascular degeneration occurs, leading to peripheral retinal vascular development stagnation or even vascular occlusion. During the hypoxic phase of ROP, the aforementioned vascular changes lead to retinal ischemia and trigger the release of growth factors, leading to pathological neovascularization. At present, the focus of ROP treatment is reducing the proliferation of new blood vessels, and the side effects of treatment, such as pain and inflammation, are also significant. However, alleviating vascular occlusion during hyperoxia can help alleviate vascular proliferation caused by subsequent ischemia and hypoxia. Therefore, we attempted to further improve knowledge of the pathogenesis of ROP during the hyperoxia period, starting from the factors and mechanisms of action to prevent it or administer early treatment.

By analyzing the differential expression factors of ROP patients, this study revealed that CXCL10 was highly expressed in ROP patients. CXCL10 is a proinflammatory cytokine that binds to CXCR3 and engages in various G protein-mediated signal transduction processes, including peripheral immune cell chemotaxis, differentiation and activation, cell growth regulation, apoptosis regulation, and vascular inhibition in various cells (Xia et al. 2016; Romagnani et al. 2001; Sidahmed et al. 2012; Kong et al. 2021; Smit et al. 2003; Gao et al. 2009). Previous studies have shown that the CXCL10/CXCR3 axis exacerbates the migration of pulmonary artery adventitia fibroblasts and participates in the formation of vascular embolic substances (Hong et al. 2022). Therefore, we hypothesize that after undergoing external stimulation, the secretion of CXCL10 by endothelial cells increases, leading to an increase in CXCL10/CXCR3 binding and exacerbating endothelial dysfunction, thereby exacerbating vascular occlusion.

Ferroptosis is a leading factor in various ischemia–reperfusion injuries across different organs, including stroke, cardiovascular ischemia, ischemia/reperfusion-induced acute kidney injury, and liver disease, among others. It plays a crucial role in vascular occlusion due to the death of retinal endothelial cells. Unlike apoptosis and necrosis, this distinct form of cell death is characterized by significant iron accumulation and lipid peroxidation (Vähätupa et al. 2023; Li et al. 2020).

When lipid metabolism is abnormal and ROS are produced, the excessive accumulation of iron ions in endothelial cells can lead to ferroptosis, resulting in vascular occlusion (Vähätupa et al. 2023; Li et al. 2020; Lin et al. 2022a). Astrocytes can increase the phosphorylation of signal transducer and activator of transcription 3 (STAT3) through the secretion of CXCL10 and inhibit solute carrier family 7 member 11 (SLC7A11) in neurons through CXCR3, thereby exacerbating ferroptosis (Liang et al. 2023). Therefore, we speculated that the CXCL10/CXCR3 axis induces ferroptosis in endothelial cells, leading to vascular occlusion. Fer-1 is a ferroptosis inhibitor, while Erastin is a ferroptosis inducer. We applied Fer-1 and Erastin to treat vascular endothelial cells to simulate an environment that inhibits and induces ferroptosis, which helps to explore the relationship between ROP and ferroptosis. We also used AMG-487, a CXCR3 inhibitor, to block CXCL10/CXCR3 binding to observe whether curcumin inhibits ferroptosis via the CXCL10/CXCR3 axis.

We explored the efficacy of curcumin using CXCL10 as a disease target, as curcumin is strongly related to CXCL10. Curcumin, a curcuma derivative, is a lipophilic polyphenol that has been employed in in vivo studies and clinical trials as an anticancer and anti-inflammatory medication (Kotha and Curcumin 2019; Anand et al. 2008); it can inhibit CXCL10 expression and reverse ferroptosis to protect cells (Zhang et al. 2022a; Tu et al. 2011). However, the effect of curcumin on retinal blood vessels needs to be further explored. Therefore, in this study, the expression of differentially expressed factors was explored through the Gene Expression Omnibus (GEO) database, and an animal (OIR) and cell model were constructed to verify their expression. CoIP was used to investigate the binding partners of CXCR3 and ferroptosis-related proteins, and molecular docking and validation experiments were used to determine the vascular protection effect and the inhibitory effect of curcumin on ferroptosis. The findings from this study lay the foundation for providing new ideas for the treatment of ROP.

## Methods

### GEO database analysis

In the GEO database, we searched for information about ROP patients (GSE32472). Premature delivery at a gestational age of less than 32 weeks and a birth weight of 1500 g were the inclusion criteria. Blood samples from normal children and children with ROP at 2 weeks after birth were labeled N and R, respectively. Blood samples from patients with ROP at 0 days and 2 weeks after birth were annotated as RA and RB, respectively. FC represents the ratio of the abundance of a particular factor in the ROP group to its abundance in the normal group. To identify differentially expressed variables, a P value of 0.05 and an FC value > 1 were used as screening criteria. For the Protein–Protein Interaction (PPI) network, data were imported from the Search Tool for Recurring Instances of Neighboring Genes (STRING) database, a search engine for interacting genes, into Cytoscape software to create an interactive network. The Basic Local Alignment Search Tool (BLAST) program was used to compare gene sequences to the Kyoto Encyclopedia of Genes and Genomes (KEGG) database, after which pathway annotation information for all genes was extracted. The online analysis website Metascape (https://metascape.org/gp/index.html#/main/step1) was used to perform KEGG enrichment analysis on the differentially expressed factors. All relevant factors related to ferroptosis were identified using the ferroptosis-significant gene pool (http://www.zhounan.org/ferrdb/current/).

The lung tissues of normal and hyperoxic C57 mice (GSE51039) were compared, and the differentially expressed factors were selected on the basis of *P* < 0.05. Overlap analysis of ferroptosis-related factors was performed, and Pearson rank correlation was used to test the factors significantly correlated with CXCL10.

Changes in the enrichment levels of different factors in normal and hydrogen peroxide (H_2_O_2_) stimulated vascular endothelial cells (GSE72991) were assessed at 0 h, 2 h, 4 h, 6 h, 8 h, 10 h, 12 h, 14 h and 16 h.

### Cell culture

Human retinal microvascular endothelial cells (HRMECs) were grown in complete DMEM (Gibco BRL, Gaithersburg, MD, USA), 10% fetal bovine serum (Gibco BRL, Gaithersburg, MD, USA), 10,000 U/ml penicillin, and 10,000 g/ml streptomycin (Hyclone, Logan, UT, USA) at 37 °C in a 5% CO_2_ incubator. For experiments, cells from the third passage were used.

Prepare curcumin (Glpbio, Montclair, CA, USA) as a liquid reagent with a concentration of 200 mM using DMSO (Solarbio, Beijing, China) and store it for future use. Subsequently, DMSO was applied to dilute it to 8 mM liquid and added to the culture medium according to the required concentration, ensuring that the DMSO content in each milliliter of culture medium is less than or equal to 1 μl.

The cells were divided into a blank control group (NC) (0.1% DMSO, 12 h) (Solarbio, Beijing, China), ferroptosis inhibition group (Fer-1, 20 μM, 12 h) (Selleck, Shanghai, China), ferroptosis group (Erastin, 5 μM, 12 h) (Selleck, Shanghai, China), H_2_O_2_ group (H_2_O_2_, 400 μM, 12 h) (Sigma, St Louis, MO, USA), H_2_O_2_ + curcumin group (H + C) (curcumin, 2 μM, 12 h) (Glpbio, Montclair, CA, USA), Erastin + curcumin group (E + C), CXCR3 inhibition group (AMG-487) (AMG-487, 0.5 μM, 12 h) (MCE, NJ, USA), Erastin + AMG-487 group (E + A), and H_2_O_2_ + AMG-487 group (H + A).

### Assessment of cell viability

MTT (Solarbio, Beijing, China) was used following the manufacturer's instructions to determine cell viability. The experiment was repeated three times using a 96-well plate with 3 wells for each group. After culturing each batch of cells, the media was removed, and 10 μl of MTT (5 mg/ml) was added to each well of the cell culture plate. After four hours of incubation at 37 °C, the supernatant was discarded. Then, 150 μl of DMSO was added to each well, and the plate was oscillated for 10 min. A microplate reader (Thermo 3001 VARIOSKAN FLASH, CA, USA) was used to determine the absorbance at a wavelength of 490 nm.

### Real-time quantitative PCR

RNA was extracted as directed (EZ bioscience, USA), and the concentration was measured with a NANODROP 2000 spectrophotometer (Thermo Fisher Scientific, Waltham, MA, USA). A reverse transcription reagent (Abm, Richmond, BC, Canada) was applied to convert the RNA into cDNA in a 20-µl system. Reaction mixture (diluted cDNA, forward and reverse primers, SYBR Green FastStart 2 × Master Mix (Abm, Richmond, BC, Canada)) was added to the wells of a 96-well plate (Labselect, Beijing, China). The qPCR program was as follows: 95 °C for 3 min; and 40 cycles of 95 °C for 15 s and 60 °C for 1 min. Amplification was performed on a qPCR instrument (Roche LightCycler 96, Switzerland). The relative expression level of the cathepsin gene was determined using the comparative threshold cycle (2^−∆∆Ct^) (Table 1).

**Table 1:** Primer sequences

Gene	Species	PubMed ID	Sequence (5’ → 3’)
β-actin	Human	NM_001101.5	F: CCTTCCTTCCTGGGCATGG
			R: TCTGCATCCTGTCGGCAATG
CXCR3	Human	NM_001142797.2	F: TTTGACCGCTACCTGAACATAGT
			R: GGGAAGTTGTATTGGCAGTGG
CXCL10	Human	NM_001565.4	F: GGAGGATGGCAGTGGAAGTC
			R: GTGGATGTTCTGACCCTGCT
TFRC	Human	NM_003234.4	F: TCGGAGAAACTGGACAGCAC
			R: ATCACGCCAGACTTTGCTGA
HIF1-α	Human	NM_001243084.2	F: GAACGTCGAAAAGAAAAGTCTCG
			R: CCTTATCAAGATGCGAACTCACA
STAT3	Human	NM_001384993.1	F: CAGCAGCTTGACACACGGTA
			R: AAACACCAAAGTGGCATGTGA
PTGS2	Human	NM_000963.4	F: AGGCTTCCATTGACCAGAGC
			R: TCCACAGCATCGATGTCACC
TXN	Human	NM_003329.4	F: CGTGGCTGAGAAGTCAACTACTA
			R: GTGAAGCAGATCGAGAGCAAG
VCP	Human	NM_001354927.2	F: GATCAGGAGCCAGCGTTGTT
			R: CTTTTGAACCTGCGCGGC
HSPB1	Human	NM_001540.5	F: GAGCTGACGGTCAAGACCAA
			R: TGGTGATCTCGTTGGACTGC
GAPDH	Mus	NM_001411843.1	F: TGGCCTTCCGTGTTCCTAC
			R: GAGTTGCTGTTGAAGTCGCA
CXCR3	Mus	NM_009910.3	F: GCCTCAATCCGCTGCTCTAT
			R: ATGCTGAGCTGTCAGTGCAT
CXCL10	Mus	NM_021274.2	F: CGCTGCAACTGCATCCATATC
			R: TAGGCTCGCAGGGATGATTTC
TFRC	Mus	NM_011638.4	F: TGAGTGGCTACCTGGGCTAT
			R: CTCCTCCGTTTCAGCCAGTT
HIF1-α	Mus	NM_010431.3	F: ACCTTCATCGGAAACTCCAAAG
			R: CTGTTAGGCTGGGAAAAGTTAGG
FTH1	Mus	NM_010239.2	F: GGCTGAATGCAATGGAGTGTG
			R:CTCAATGAAGTCACATAAGTGGGG

### Measurement of malondialdehyde (MDA)

HRMECs and mice retinal tissue were collected for total protein extraction. The protein concentration of samples was previously determined using a BCA assay kit (Thermo Fisher Scientific, Waltham, MA, USA). An MDA assay kit (Nanjing Jiancheng, Nanjing, China) was used to assess lipid peroxidation. The blank, standard, sample, and reference tubes were set up as directed. The sample was mixed with a vortex mixer for 10 min, placed in a 95 °C water bath (or boiled with the lid open) for 40 min, cooled under running water, and then centrifuged at 3500–4000 rpm for 10 min (the centrifugation time must be extended for centrifugations below 3000 rpm to accomplish complete sedimentation). The absorbance of the supernatant was measured at 532 nm with a microplate reader (Thermo 3001 VARIOSKAN FLASH, CA, USA). The MDA content was calculated with the following formula: (measured OD value—blank OD value)/(standard OD value-blank OD value) *standard concentration (10 nmol/ml)/protein concentration (mgprot/ml).

### Measurement of glutathione (GSH) levels

HRMECs and mice retinal tissue were collected for total protein extraction. The protein concentration of samples was previously determined using a BCA assay kit. A GSH test kit (Nanjing Jiancheng, Nanjing, China) was used to perform a quantitative analysis of reduced glutathione expression (built in Nanjing, Nanjing, China). The appropriate chemicals were added to the blank, standard, and sample wells. A microplate reader (Thermo 3001 VARIOSKAN FLASH, California, USA) was used to assess the absorbance of each well at 405 nm after mixing and standing. The GSH concentration in tissues and cells was calculated as follows: (measured OD value-blank OD value)/(standard OD value—blank OD value) *standard concentration (20 μM) *dilution ratio/protein concentration (gprot/L).

### ROS

Cells were seeded at a density of 2 × 10^4^ per well in a 24-well plate. The cell model was created 12 h after the cells adhered to the wells. Subsequently, the supernatant was discarded, and 200 µl (1:1000 serum-free medium) of diluted DCFH-DA (Nanjing Jiancheng, Nanjing, China) was added to each well. The cells were incubated for 30 min and washed with serum-free medium three times before being observed under a fluorescence microscope.

### Live/dead cell staining

Live/Dead Cell Double Staining Kit (Solarbio, Beijing, China) is used to detect cell viability. After stimulation, the cells from each group were collected in centrifuge tubes, resuspended in 1 × Assay Buffer, mixed with 2 μl of calcein AM per 1 ml of cell volume, and incubated in the dark at 37 °C for 25 min. Then, 5 μl of PI was added for 5 min. After centrifugation to remove the staining solution, the cells were resuspended in PBS. The solution was dropped onto a glass slide, and photos were taken using a confocal microscope (Leica TCS SP8, Heidelberg, Germany).

### Transwell cell migration assays

Serum-free culture media (100 μl) was added to the upper chamber of Transwell inserts (8 μm, Costar, USA), which were then placed in a cell incubator for 30 min to humidify and preheat. During this period, cells in T25 flasks were digested to create a cell suspension. After 30 min, the Transwell chamber was removed from the cell culture incubator, 750 μl of culture medium containing 20% serum was added to the lower chamber, and 200 μl of the cell suspension was added to the upper chamber. Then, the cells were placed in a cell incubator for 16 h and then washed with PBS, fixed with 4% paraformaldehyde (Solarbio, Beijing, China) for 30 min, and stained with crystal violet (Beyotime, Shanghai, China). After staining, the surface of the chamber was gently wiped with a cotton swab to remove cells. After drying, a sterile sharp knife was used to cut off the bottom surface of the chamber, which was then sealed with neutral gum. This experiment was conducted three times, with each group having three repeated wells. For quantification, images were taken with a microscope (Olympus Corporation, Tokyo, Japan), and then, fields of view were randomly selected for each group to count the number of migrated cells. The data were recorded and saved for further statistical analysis.

### Cell scratch test

Cells were seeded at a density of 3 × 10^4^ per well in a 24-well plate. After the cells adhered to the wells, the monolayer was scraped with a pipette tip. Then, the cells were rinsed three times with PBS. After scratching the monolayer, the cells were incubated in serum-free culture medium. Fixed-point photos were taken every hour for 15 h. The results were analyzed using ImageJ software.

### Acquisition of the disease and drug target gene database and molecular docking of drugs and proteins

Genes and drug sensitivity data were used to determine the efficacy of appropriate treatments, that is, CXCL10 was used to determine the efficacy of curcumin (http://portals.broadinstitute.org/ctrp/). Targets related to curcumin were selected from the following databases: SwissTargetPrediction (http://www.swisstargetprediction.ch) and BATMAN-TCM (http://bionet.ncpsb.org.cn/batman-tcm/#/home) (Filter criteria: score > 5). Targets related to ROP were filtered from the following databases: Human GeneCards (GeneCards, https://www.genecards.org/) (Filter criteria: probability > 30), DisGeNET (https://www.disgenet.org/), and The Human Mendelian Genetic Database (OMIM, https://omim.org/). The structure was searched through the PubChem database (https://pubchem.ncbi.nlm.nih.gov). Protein structures were obtained from a protein database (https://www.rcsb.org/; UniProt, https://www.uniprot.org). Open Babel software was used to convert small molecule structures, and PyMol software was used to remove water and homologous subunits from CXCL10 and CXCR3. Then, molecular docking software (AutoDock-Vina) was used to dock curcumin with CXCL10 and CXCR3.

### Enzyme-linked immunosorbent assays

Mouse retina was thoroughly ground and then centrifuged, after which the supernatant was retained. The concentrations of CXCL10 and CXCR3 in the samples were measured using enzyme-linked immunosorbent assay (ELISA) kits (Jiangsu Jingmei, Jiangsu, China). Serially diluted standards and properly diluted samples were added to a 96-well microplate precoated with purified antibodies to bind with the target protein. After 30 min at room temperature, the solution was discarded, and the wells were carefully washed. Then, 50 μl of enzyme labeling reagent was added to the center of each well. After thorough washing, chromogenic reagents were applied. After incubating for 10 min in the dark at 37 °C, termination solution was added to stop the reaction. An Infinite 200 PRO Multimode Microplate Reader (Tecan Group Ltd., Männedorf, Switzerland) was used to measure the absorbance at 450 nm. The CXCL10 and CXCR3 protein concentrations in each sample were determined from standard curves.

### Immunofluorescence

Following tissue embedding, the samples were sliced, fixed, dewaxed, and subjected to antigen retrieval. After the sections were washed with PBS and fixed with 4% paraformaldehyde, subsequent steps were performed as described above for cells. For permeabilization, the sections were incubated in PBS containing 0.1% Triton X-100 (Solarbio, Beijing, China) for 10 min. After washing with PBS, the sections were blocked for 1 h with 1% BSA (Biotopped, Beijing, China). After overnight incubation at 4 °C, the sections were incubated with 5 antibodies: anti-CXCL10 (1:250, bs-23238r, Bioss, Beijing, China), anti-CXCR3 (1:250, bs-23781r, Bioss, Beijing, China), anti-HIF-1α (1:250, 20,960, Proteintech, Wuhan, China), anti-FTH (1:250, ab183781, Abcam, Cambridge, UK), and anti-TFR (1:250, ab214039, Abcam, Cambridge, UK). After washing with PBS, the sections were incubated for 1 h at room temperature with anti-rabbit (1:1000, ab150077, Abcam, Cambridge, UK) or anti-mouse (1:1000, ab150116, Abcam, Cambridge, UK) secondary antibodies.

### Mice and the OIR model

Prepare curcumin into a liquid reagent with a concentration of 200 mM using DMSO solution according to the instructions. In the normal group (N), no treatment was administered, and the animals were raised under normal oxygen conditions.

In the control group, the mice were raised under normal oxygen conditions and intraperitoneally injected with saline and nasally administered saline at P7, P9, and P11. Mice were sampled at P12 (recorded as P12NC) and at P17 (recorded as P17NC).

In the OIR + curcumin group, the mice were raised under high oxygen conditions from P7 to P12 and were intraperitoneally injected with curcumin (200 mM, 30 μl/g) and nasally administered curcumin (200 mM, 10 μl) at P7, P9, and P11. Mice were sampled at P12 (recorded as P12R + C_P7-12_). P17R + C_P7-12_ mice were subsequently raised under normal oxygen conditions from P12 to P17. Mice sampled at P17 (recorded as P17R + C_P12-17_) were placed in a high oxygen chamber from P7 to P12 but without drug injection but with the intraperitoneal injection of curcumin (200 mM, 30 μl/g) and nasal administration of curcumin (200 mM, 10 μl) under normal oxygen conditions from P12 to P17 (Fig. Supplementary 1).

### Retinal preparation

After anesthesia with chloral hydrate, the eyeballs were removed, soaked in 4% paraformaldehyde (Solarbio, Beijing, China) at room temperature for 2 h. Under a surgical microscope, the intact retina was dissected, and each retina was cut into four sections. The sections were then washed with PBS. The sections were blocked and permeabilized in PBS supplemented with 3% BSA (Biotopped, Beijing, China) and 0.1% Triton X-100 (Solarbio, Beijing, China) for one hour at 4 °C. The retina sections were then incubated with anti-IB4 (1:50) (Maokang Biology, Shanghai, China) and anti-GFAP (1:200) (CST, Boston, MA, US) antibodies at 4 °C overnight. After washing, the retina sections were fixed on glass slides, rinsed with PBS, and incubated with a secondary antibody. The sections were visualized under a confocal microscope (Leica TCS SP8, Heidelberg, Germany).

### Quantitative evaluation of the retinal vascular network

The ischemic area of the retina was calculated using ImageJ 2.1.0 software, and then, the percentages of the ischemic and neovascularized areas were recorded and compared to those of the overall retina. High-resolution photos of the avascular retina were collected, and the number of tip cells in the ischemic portion of the retina, as well as the ratio of distal sprouts or filopodia per 1 mm of vascular length in each field of view, were determined. The form and number of astrocytes in the ischemic and neovascular areas of the retina were analyzed.

### Mouse weighing

The mice were weighed after they were placed in a small dish.

### CoIP with mass spectrometry

After centrifugation, cells in a T25 flask were digested, and the cells were placed in NP-40 to generate protein lysates (Solarbio, Beijing, China). After centrifugation, the complete tissue protein was collected. To determine the protein concentration, a BCA assay kit (Solarbio, Beijing, China) was used according to the manufacturer's instructions.

A portion of each sample (as an input control) was removed, and 50% agarose beads was added to 300 μL of sample. The sample was mixed well at 4 °C and centrifuged for 5 min, after which the supernatant was collected. An anti-CXCR3 antibody was added to 200 μl of supernatant, 3.5 μl of control IgG was added to 100 μl of supernatant, and the samples were rotated and mixed overnight at 4 °C. Agarose beads were added to the IgG control group and IP group to capture antigen–antibody complexes. The beads were washed with precooled PBS, and the supernatant was collected by centrifugation. The agarose beads containing the antigen–antibody complex were boiled, the antigens, antibodies, and beads were separated, and the supernatants were collected.

Qualitative proteomics was performed on the collected samples. Paraffin-embedded samples were cut with a scalpel into small pieces (approximately 1 mm^3^). Each sample was placed in a 1.5-mL centrifuge tube, decolorizing solution was added, and the tube was shaken at a constant speed; this process was repeated multiple times until the sample was decolorized and transparent.

Acetonitrile was added to dehydrate the samples until colloidal granules turned white. Then, each sample was vacuum-dried. DTT working solution (Amresco, Shanghai, China) was added to a final concentration of 10 mM, followed by incubation at 37 °C for 1 h. Then, acetonitrile was added to dehydrate the samples until the colloidal granules turned white. After vacuum drying, IAM working solution (Amresco, Shanghai, China) was added to a final concentration of 55 mM, and the samples were incubated in a dark room for 30 min. Acetonitrile (J.T.Baker, Anaheim, CA, USA)was added to dehydrate each sample until colloidal granules turned white. The samples were vacuum-dried, and deionized water was used to wash the samples. This step was repeated once. Then, 50 mM ammonium bicarbonate (Sigma, St Louis, MO, USA) was added, and the samples were incubated for 10 min, after which trypsin working solution was added. The enzyme solution was allowed to come into full contact and be completely absorbed by the samples, followed by incubation overnight at 37 °C. The supernatant was collected by centrifugation and placed in a new centrifuge tube; acetonitrile was added to the remaining samples, followed by vortexing for 5 min. The supernatant was collected by centrifugation and combined with the supernatant from the previous step. Formic acid (FA) (0.1%) (Sigma, St Louis, MO, USA)was added to the remaining samples. After absorbing the liquid, acetonitrile was added, and the sample was vortexed for 5 min. The supernatant was collected and combined with the supernatant collected in the previous steps. The sample was freeze-dried under vacuum. The samples were desalted using a C18 desalting column activated with 100% acetonitrile. The column was equilibrated with 0.1% formic acid, after which the samples were loaded onto the column. Subsequently, the column was washed with 0.1% formic acid to remove impurities and finally eluted with 70% acetonitrile. The efflux was collected and lyophilized.

The mobile phases, i.e., liquid A (100% water, 0.1% formic acid) and liquid B (80% acetonitrile, 0.1% formic acid), were prepared. The freeze-dried powder was dissolved in 10 µl of solution A and centrifuged at 14,000× *g* for 20 min at 4 °C. One microgram of each supernatant sample was collected for quality assessment with liquid chromatography elution strips. An Orbitrap Exploris™ 480 mass spectrometer was used, and the FAIMS Pro™ Interface was used. The compensation voltage (CV) switched every 1 s between – 45 and – 65. The ion spray voltage was set to 2.0 kV, and the temperature of the ion transfer tube was set to 320 °C. Data-dependent acquisition mode was adopted for mass spectrometry. The full scan range of mass spectrometry was m/z 350–1500, with a primary mass spectrometry resolution of 120,000 (200 m/z), an AGC of 300%, and a maximum injection time of 50 ms for the C-trap. For secondary mass spectrometry detection, "Top Speed" mode was used, with a resolution of 15,000 (200 m/z), an AGC of 75%, a maximum injection time of 22 ms, and a peptide fragmentation collision energy of 33% to generate the raw mass spectrum detection data. The *Homo sapiens* sp. database was used, and Proteome Discoverer 2.4 software was used.

### Statistical analysis

All the results were obtained from at least three independent experiments. SPSS 26.0 software was used for statistical analyses. The data are presented and plotted as the mean ± standard deviation (GraphPad Prism software). The D'Agostino Pearson normality test was used to test the distribution of the data. Independent sample t tests were performed to compare two groups, and one-way ANOVA was used to compare multiple samples. *P* < 0.05 was regarded as significant.

## Results

### CXCL10 is closely linked with ferroptosis in ROP patients according to GEO data

**Fig. 1:**
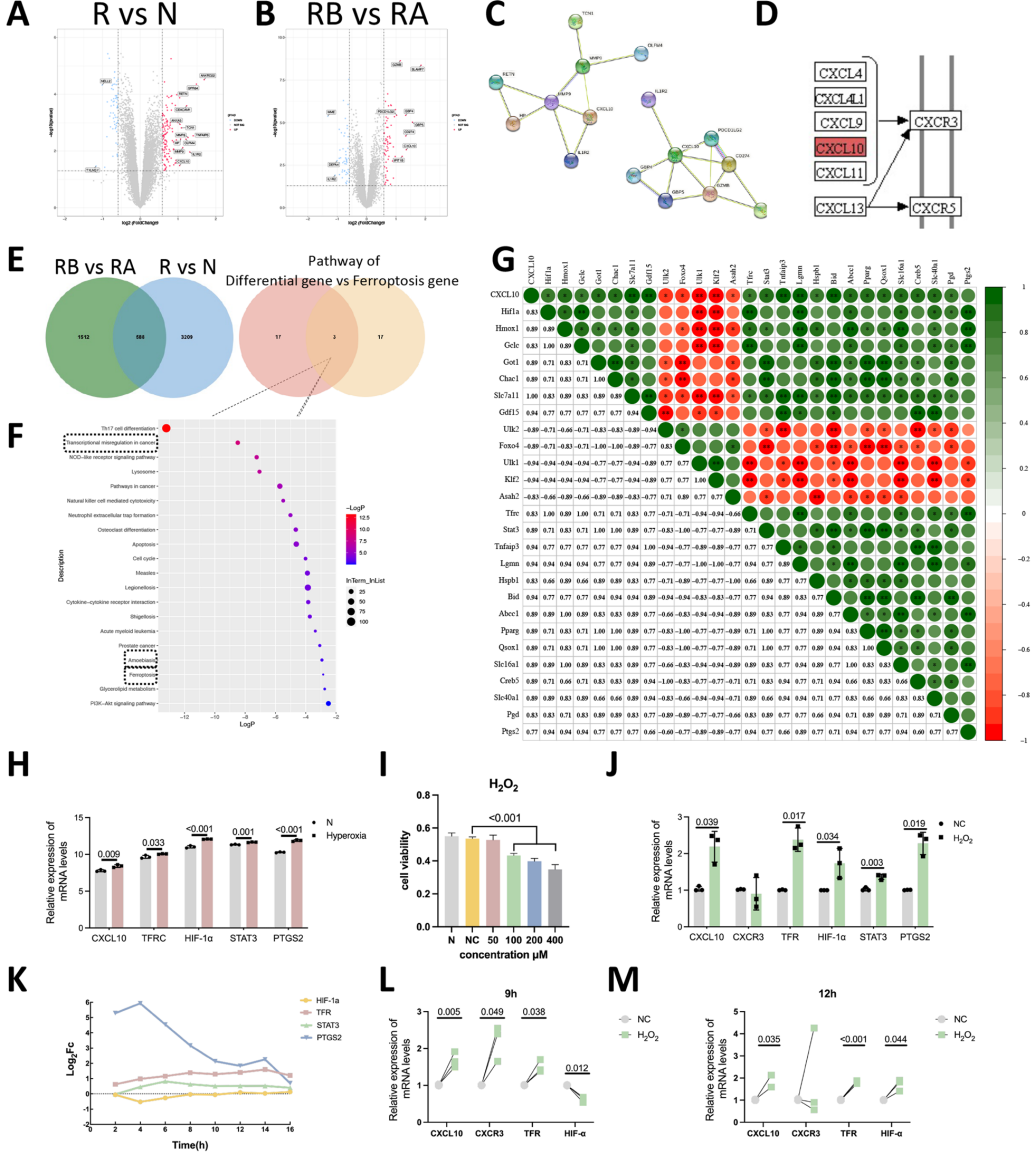
GEO data reveals differential CXCL10 expression in ROP linked to ferroptosis. **A** Volcano plot demonstrating significant differences in gene expression between the R and N groups. Differentially expressed genes with *P* values < 0.05 and |logFC|> 1 are labeled in the graph. **B** Volcano map of differentially expressed genes between the RB and RA groups. Differentially expressed genes with *P* values < 0.05 and |logFC|> 1 are labelled in the graph. **C** Interactions between differentially expressed components in the RA and RB groups and in the N and R groups. **D** KEGG pathway analysis revealed that the CXCL10 receptor is CXCR3. **E** RA vs. RB and N vs. R Venn diagrams revealed 588 similar differentially expressed components, with CXCL10 expression overlapping. Differential gene and ferroptosis-related gene Venn diagrams revealed 3 repetitive expression pathways. **F** KEGG pathway enrichment of all factors related to ferroptosis. Three pathways, i.e., ferroptosis, amoebiasis, and transcriptional dysregulation in cancer, overlapped, as shown in **E**. **G** TFR, HIF-1α, STAT3, and PTGS2 in the ferroptosis pathway are significantly correlated with CXCL10. **H** CXCL10, TFR, HIF-1α, STAT3 and PTGS2 expression is upregulated in the lung tissue of hyperoxic mice. **I** Changes in cell viability after stimulation with different concentrations of H_2_O_2_. **J** Different factors were differentially expressed in the NC and H_2_O_2_ groups. **K** Changes in the expression of various factors with different H_2_O_2_ stimulation durations. **L**, **M** Different factors were differentially expressed in the normal group, the group stimulated with H_2_O_2_ for 9 h and 12 h

Data from ROP patients in the GEO database (GSE32472) revealed that Killer cell lectin like receptor C4 (KLRC4), Ankyrin repeat domain 22 (ANKRD22), G protein-coupled receptor 84 (GPR84), Neural EGFL like 2 (NELL2), Fc fragment of IgG receptor Ic (FCGR1A), Resistin (RETN), Carcinoembryonic antigen related cell adhesion molecule 1 (CEACAM1), Annexin A3 (ANXA3), Transcobalamin 1.

(TCN1), TNF alpha induce haptoglobind protein 6 (TNFAIP6), Matrix metallopeptidase 8 (MMP8), Haptoglobin (HP), Olfactomedin 4 (OLFM4), Matrix metallopeptidase 9 (MMP9), Interleukin 1 receptor type 2 (IL1R2), CXCL10, and Taxilin gamma Y-linked (pseudogene) (TXLNGY) were differentially expressed genes between the R and N groups (Fig. 1A). The expression of granzyme B (GZMB), Contactin associated protein-like 3B (CNTNAP3), SLAM family member 7 (SLAMF7), Imprinted maternallyexpressed transcript (H19), Programmed cell death 1 ligand 2 (PDCD1LG2), Guanylate binding protein 4 (GBP4), Membrane metallo-endopeptidase (MME), Guanylate binding protein 1 (GBP1), Guanylate binding protein 5 (GBP5), CD274 molecule (CD274), CXCL10, Interferon induced protein with tetratricopeptide repeats 1B (IFIT1B), Defensin alpha 4 (DEFA4), and Interleukin 1 receptor type 2 (IL1R2) was upregulated in the RA and RB groups (Fig. 1B). After conducting PPI analysis on two groups of significantly different factors, it was found that CXCL10 was differentially expressed in both groups and played a major pivotal role (Fig. 1C). We believe that CXCL10 plays an important role in the early progression of ROP; therefore, we chose CXCL10 for further research. KEGG pathway analysis revealed that CXCL10 acts by binding to the CXCR3 receptor (Fig. 1D).

In a comparison of the differentially expressed genes (DEGs) between the R vs. N group and RB vs. RA group, there were 588 DEGs (Fig. 1E). KEGG pathway enrichment analysis of these 588 genes revealed that the DEGs were enriched in the ferroptosis pathway (Fig. 1E). This finding is consistent with vascular occlusion caused by ferroptosis during the hyperoxic phase of ROP(Wang et al. 2022a). Subsequently, pathway enrichment analysis of factors related to ferroptosis revealed that three pathways, namely, ferroptosis, amoebic disease, and transcriptional dysregulation in cancer, were enriched in both groups (Fig. 1F). The ferroptosis pathway is more relevant to our research; therefore, we chose this pathway for further research.

The expression of various factors in the lung tissue of normal and hyperoxia-stimulated mice in the GEO database was compared, and the differentially expressed factors related to ferroptosis were explored. Transferrin receptor (TFR), Hypoxia-inducible factor 1 subunit alpha (HIF-1α), STAT3, and Prostaglandin-endoperoxide synthase 2 (PTGS2) in the ferroptosis pathway were significantly correlated with CXCL10 (r = 0.829, 0.829, 0.886, 0.771; *P* = 0.042, 0.042, 0.019, 0.072) (Fig. 1G). Moreover, CXCL10, TFR, HIF-1α, STAT3 and PTGS2 expression was upregulated in the lung tissue of hyperoxic mice (Fig. 1H). This finding is consistent with our expectations, and we speculate that CXCL10 and ferroptosis play roles in the hyperoxic phase of ROP.

H_2_O_2_ was used to generate an in vitro retinal vascular damage model caused by oxidative stress. According to the MTT findings, after 12 h of treatment, the viability of cells treated with 100 and 200 μM H_2_O_2_ significantly decreased, and treatment with 400 μM H_2_O_2_ greatly increased cytotoxicity (Fig. 1I). The ideal H_2_O_2_ concentration for inducing damage in HRMECs was determined to be 400 μM.

Compared with those in the control group, the expression levels of CXCL10, TFR, HIF-1α, STAT3, and PTGS2 were higher in the H_2_O_2_ group (Fig. 1J). HIF-1α has been shown to inhibit ferroptosis (Lin et al. 2022b); however, its expression was significantly increased in the H_2_O_2_ group. A database analysis of the effects of H_2_O_2_ stimulation on HRMECs revealed that the relative difference in TFR expression increased significantly with time; however, with increasing TFR mRNA expression, the expression of HIF-1α decreased significantly at first and then increased gradually at 8–16 h (Fig. 1K). HIF-1 is the hub of multiple pathways, considering that when TFR expression increases, it can provide negative feedback to suppress HIF-1α expression (Fig. 1L), while as TFR increases, the activation of other HIF-1 pathways, such as vascular endothelial growth factor (VEGF), increases to resist the damage to cells caused by excessive TFR expression (Fig. Supplementary 2). Therefore, in this study, we compared the expression of different factors after 9 h and 16 h of H_2_O_2_ stimulation. Compared to that in the control group, the expression of CXCL10, CXCR3, and TFR was significantly higher in the 9 h H_2_O_2_ stimulation group (*P* = 0.005, 0.049, 0.038), and the expression of HIF-1α was significantly lower (*P* = 0.012) (Fig. 1L). Compared to that in the control group, the expression of CXCL10, TFR and HIF-1α was significantly higher in the 12 h H_2_O_2_ stimulation group (*P* = 0.035, < 0.001, 0.044), and the expression of CXCR3 was lower but not significantly different (Fig. 1M). Therefore, we believe that the change in HIF-1α expression is related to the degree or duration of TFR expression. The expression of CXCR3 is closely related to ferroptosis, but with increasing stimulation time, CXCR3 expression may gradually decrease due to the negative feedback inhibition of TFR. Considering the consistency with the findings in animal models, 12 h of stimulation was selected to construct the relevant model.

### CXCL10/CXCR3 exacerbates ferroptosis

**Fig. 2:**
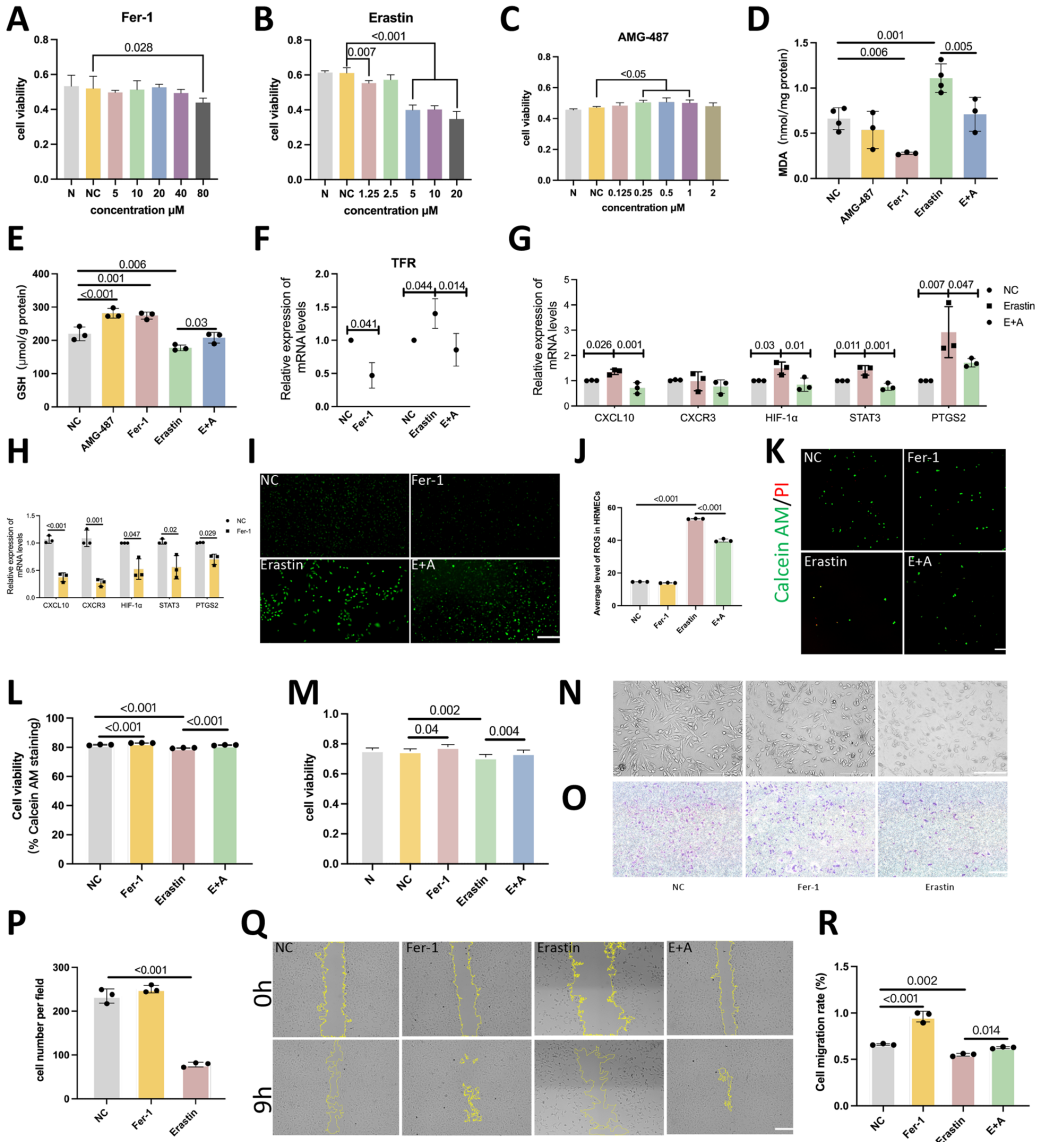
The CXCL10/CXCR3 axis exacerbates ferroptosis. **A**–**C** MTT was used to examine changes in cell viability after stimulation with different concentrations of Fer-1, Erastin, and AMG-487. **D**, **E** Changes in MDA and GSH mRNA expression in the NC, AMG-487, Fer-1, Erastin, and E + A groups. **F** TFR mRNA levels in the NC, Fer-1, Erastin, and E + A groups. **G** Different factors were differentially expressed in the NC, Erastin, and E + A groups. **H** Different factors were differentially expressed in the NC and Fer-1 group. **I** Fluorescence results for ROS in cells in the NC, Fer-1, Erastin, and E + A groups (scale bar: 200 μm). **J** Quantification of the average fluorescence intensity of ROS in cells. **K** Fluorescence images of live and dead cells in the NC, Fer-1, Erastin, and E + A groups; green fluorescence: live cells; red fluorescence: dead cells (scale bar: 25 μm). **L** Quantification of the average fluorescence intensity of live cells. **M** Quantitative comparison of the cell proliferation capacity in each group. **N** HRMEC morphology photos were captured using an optical microscope. The cell state was changed by ferroptosis. **O** Changes in the number of migrating cells in the NC, Fer-1, and Erastin groups (scale bar: 200 μm). **P** Quantitative analysis of changes in the number of migrating cells in the different groups. **Q** Changes in cell migration regions in each group at 0 h and 9 h (scale bar: 200 μm). **R** The ratio of the migration area to the original area in each group was quantified to evaluate the migration of the cells in each group

HRMECs were chosen to mimic the condition of retinal endothelial cells in patients. Fer-1 is a ferroptosis inhibitor, while Erastin is a ferroptosis inducer. To replicate the environment for preventing ferroptosis and ferroptosis, cell viability was assessed after treatment with various concentrations of Fer-1 and Erastin, and 20 μM and 5 μM, respectively, were chosen as the ideal concentrations for induction based on the MTT results (Fig. 2A, B). AMG-487 was used to block the interaction between CXCL10 and CXCR3. The cell viability was assessed after treatment with various concentrations of AMG-487 (Fig. 2C). In the AMG-487 group, the cells were treated with AMG-487 alone, and in the E + A group, the cells were treated with a combination of Erastin and AMG-487. Then, the changes in ferroptosis were evaluated (Fig. 2D–E). The Fer-1 group showed a decrease in MDA content and an increase in GSH content. The Erastin group displayed the opposite pattern, indicating the successful establishment of the model (Fig. 2D–E). Compared with those in the Erastin group, the MDA in the E + A group were lower and GSH levels in the E + A group were greater, indicating that inhibiting the interaction between CXCL10 and CXCR3 may inhibit ferroptosis (Fig. 2D–E). The trend of the qPCR results was consistent with the results for GSH and MDA. TFR mRNA expression in the E + A group was lower than that in the Erastin group, indicating decreased ferroptosis (Fig. 2F).

Compared with those in the control group, the expression levels of CXCL10, HIF-1α, STAT 3 and PTGS2 were significantly greater in the Erastin group, while the expression levels of various factors in the E + A group were lower (Fig. 2G); the expression levels of CXCL10, CXCR3, HIF-1α, STAT 3 and PTGS 2 were lower in the Fer-1 group (Fig. 2H). Consequently, the levels of CXCL10, HIF-1α, STAT3, and PTGS2 vary with ferroptosis, and the regulation of the CXCL10/CXCR3 axis also influences ferroptosis. Compared with that in the control group, the average fluorescence intensity of ROS in the Erastin group was significantly greater (P < 0.001), while it was lower in the Fer-1 group. However, compared with that in the Erastin group, the average fluorescence intensity of ROS in the E + A group was significantly lower (P < 0.001) (Fig. 2I, J). This finding suggested that intracellular ROS accumulation occurs after ferroptosis intensifies and that blocking the CXCL10/CXCR3 axis reduces intracellular ROS accumulation. The staining results for live and dead cells were consistent with these results (Fig. 2K, L). This indicates that, after ferroptosis intensifies, cell death increases and that blocking the CXCL10/CXCR3 axis reduces cell death. The results of the cell viability experiment were consistent with those described above. The number of cells in the Fer-1 group increased (*P* = 0.04), the number of cells in the Erastin group decreased (*P* = 0.002), and the number of cells in the E + A group significantly increased (*P* = 0.004) (Fig. 2M). This indicates that ferroptosis significantly inhibits cell proliferation and that blocking the CXCL10/CXCR3 axis improves cell proliferation.

Ferroptosis altered the cell state (Fig. 2N), and compared with those in the NC group, the cells in the Erastin group exhibited minor central protrusions and blurred peripheries. In the E + A group, there was an increase in cell migration, whereas in the Erastin group, there was a decrease in cell migration, as determined by scratch and transwell assays (Fig. 2O–R).

### Curcumin binds to CXCL10 to prevent ferroptosis

**Fig. 3:**
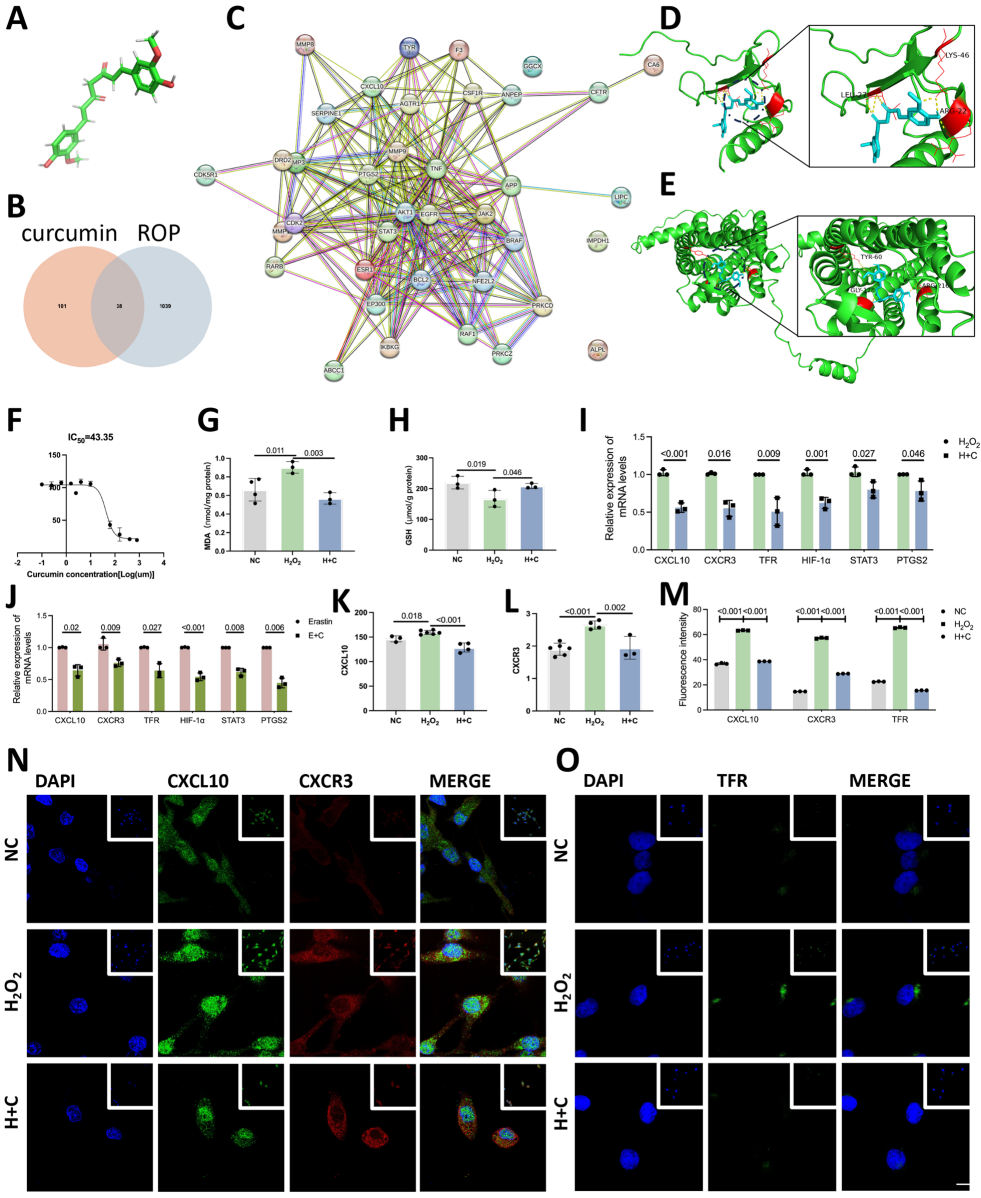
Curcumin binds to CXCL10 to prevent ferroptosis. **A** Structure of curcumin. **B** Venn diagram indicating 38 overlapping targets between the curcumin binding targets and ROP disease targets. **C** PPI interaction analysis diagram. **D** CXCL10 and curcumin have good affinity, and curcumin forms hydrogen bonds with the leucine residue at position 27, the lysine residue at position 46, and the arginine residue at position 22 in CXCL10. **E** CXCR3 has good affinity for curcumin. Curcumin forms hydrogen bonds with the tyrosine residue at position 60, glycine residue at position 128, and arginine residue at position 216 in CXCR3. **F** MTT assays revealed that the IC50 of curcumin was 43.35, indicating that the cell viability was exactly 50% when the logarithm of the curcumin concentration on the horizontal axis was 1.637. The horizontal axis represents the logarithm of the curcumin concentration, while the vertical axis represents the relative cell viability. **G** Changes in MDA levels in each group. **H** Changes in GSH expression in each group. **I** Compared with that in the H_2_O_2_ group, the expression of each factor was lower in the H + C group. **J** Compared with that in the erastin group, the expression of each factor was lower in the E + C group. **K**, **L** Comparison of CXCL10 and CXCR3 protein expression in the cell supernatants of the N, H_2_O_2_ and H + C groups. **M** Statistical chart of the average fluorescence intensity data of each group. **N**, **O** Fluorescence results for CXCL10, CXCR3 and TFR in the N, H_2_O_2_ and H + C groups (scale bar: 10 μm)

Curcumin, a natural drug, has been shown to be associated with CXCL10 (Dende et al. 2017; Yuan et al. 2017). Therefore, we conducted drug binding target experiments with curcumin based on its structure (Fig. 3A).

A total of 139 curcumin-binding targets were identified through screening. In addition, 1077 binding targets were identified for ROP. There were 38 overlapping targets of curcumin and ROP (Fig. 3B), one of which was CXCL10. Subsequently, protein interaction analysis of the 38 overlapping targets revealed that CXCL10 was strongly correlated with components linked to ferroptosis, such as PTGS2 and signal transducer and activator of STAT3, and is pivotal for ferroptosis (Fig. 3C). Curcumin binds to its protein target through hydrogen bonding and strong electrostatic interactions. CXCL10 and CXCR3 had binding energies of − 24.64 kJ/mol and − 15.73 kJ/mol, respectively (Fig. 3D, E). Curcumin can bind to both CXCL10 and CXCR3; therefore, we believe that curcumin exerts its effects by binding to CXCL10/CXCR3.

Cell viability was tested at different curcumin doses, and the IC50 value of curcumin was explored. Due to the protective effect of curcumin on blood vessels, 2 μm was chosen as the concentration for subsequent experiments. TFR mRNA expression decreased, MDA expression decreased, and GSH expression increased in the H + C group (Fig. 3G–I). This indicates that oxidative stress exacerbates ferroptosis, and this effect was reversed in the H + C group, indicating that curcumin can effectively inhibit ferroptosis. The expression of CXCL10 and CXCR3 mRNA and protein decreased in the H + C group, indicating that curcumin can inhibit the expression of CXCL10/CXCR3 (Fig. 3I, K, L). The decreased expression of HIF-1α, STAT3 and PTGS2 mRNA in the H + C and E + C groups indicated that curcumin can also regulate HIF-1α, STAT3, and PTGS2, thereby affecting CXCL10/CXCR3 (Fig. 3I, J). The immunofluorescence results supported this result (Fig. 3M–O). This finding is consistent with the results reported above, i.e., curcumin inhibited ferroptosis and relieved the ROP process by reducing the production of CXCL10 and CXCR3.

### Curcumin improves the effect of H_2_O_2_ on cell state

**Fig. 4:**
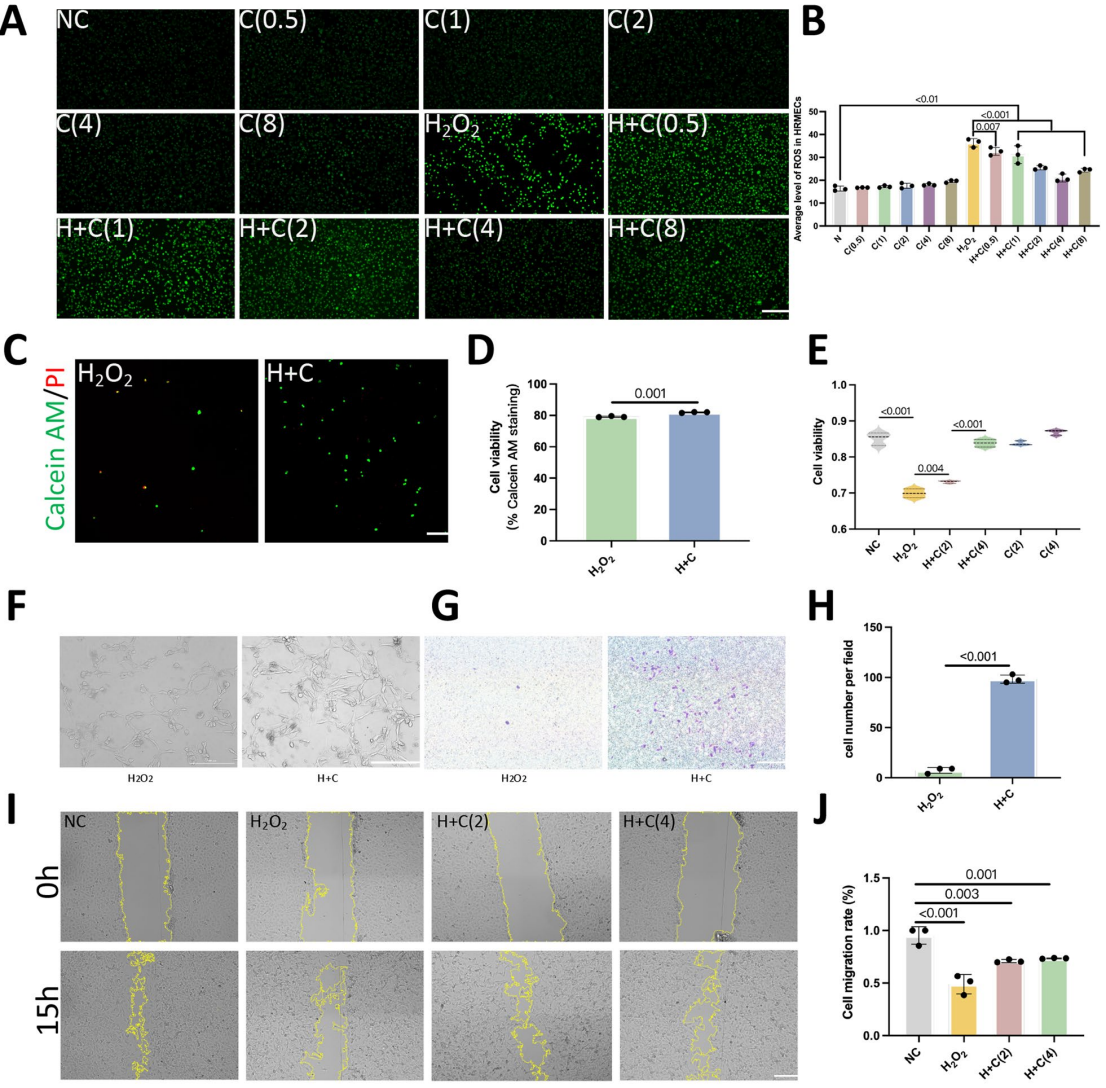
Curcumin improves cell proliferation and migration. **A** ROS fluorescence results of cells in different groups. **B** Quantification of the average fluorescence density of ROS in cells. **C** Fluorescence images of live and dead cells in the H_2_O_2_ and H + C groups; green fluorescence: live cells; red fluorescence: dead cells (scale bar: 25 μm). **D** Quantification of the average fluorescence intensity of live cells. **E** Quantitative comparison of the cell proliferative capacity in each group. **F** HRMEC morphology photos were captured using an optical microscope. The H_2_O_2_ groups showed blurred cell edges and mild central protrusions. The H + C group showed better cell adhesion and clearer morphology than did the H_2_O_2_ group. **G** Changes in the number of migrating cells in the H_2_O_2_ and H + C groups (scale bar: 200 μm). **H** Quantitative analysis of changes in the number of migrating cells in the different groups. **I** Changes in cell migration regions in each group at 0 h and 15 h (scale bar: 200 μm). **J** The ratio of the migration area to the original area in each group was quantified to evaluate the migration of the cells in each group

Compared with that in the H_2_O_2_ group, the average fluorescence intensity of ROS in the H + C (0.5 μm), H + C (1 μm), H + C (2 μm), H + C (4 μm) and H + C (8 μm) groups was significantly lower (*P* = 0.007, < 0.001, < 0.001, < 0.001, < 0.001). In the C (0.5 μm), C (1 μm), C (2 μm), C (4 μm) and C (8 μm) groups treated with curcumin alone, there was no significant change in the average fluorescence intensity (Fig. 4A, B). Compared to that in the H_2_O_2_ group, the average fluorescence intensity of the dead cells in the H + C group was significantly lower (*P* < 0.001) (Fig. 4C, D). This indicates that curcumin can alleviate H_2_O_2_-induced cell death; moreover, the results of the cell viability experiments are consistent with these findings (Fig. 4E). This indicated that the oxidative stress induced by H_2_O_2_ significantly inhibited cell proliferation and that curcumin was able to protect the cells from damage caused by oxidative stress in a dose-dependent manner. The H_2_O_2_ group showed blurred cell edges and central protrusions, and the H + C group showed better cell adhesion. Compared with that in the H_2_O_2_ group, the number of cells that crossed the membrane was significantly greater in the H + C group (*P* < 0.001) (Fig. 4G-H). The scratch assay results showed that the migration of cells in the H_2_O_2_ group significantly decreased (*P* < 0.001); adding different concentrations of curcumin improved the migration ability of cells in the H_2_O_2_ group (*P* = 0.003, 0.001) (Fig. 4I, J). All of the above findings indicate that curcumin increases cell migration.

### Curcumin decreases avascular area in OIR mice under hyperoxia and enhances physiological blood vessel sprouting

**Fig. 5:**
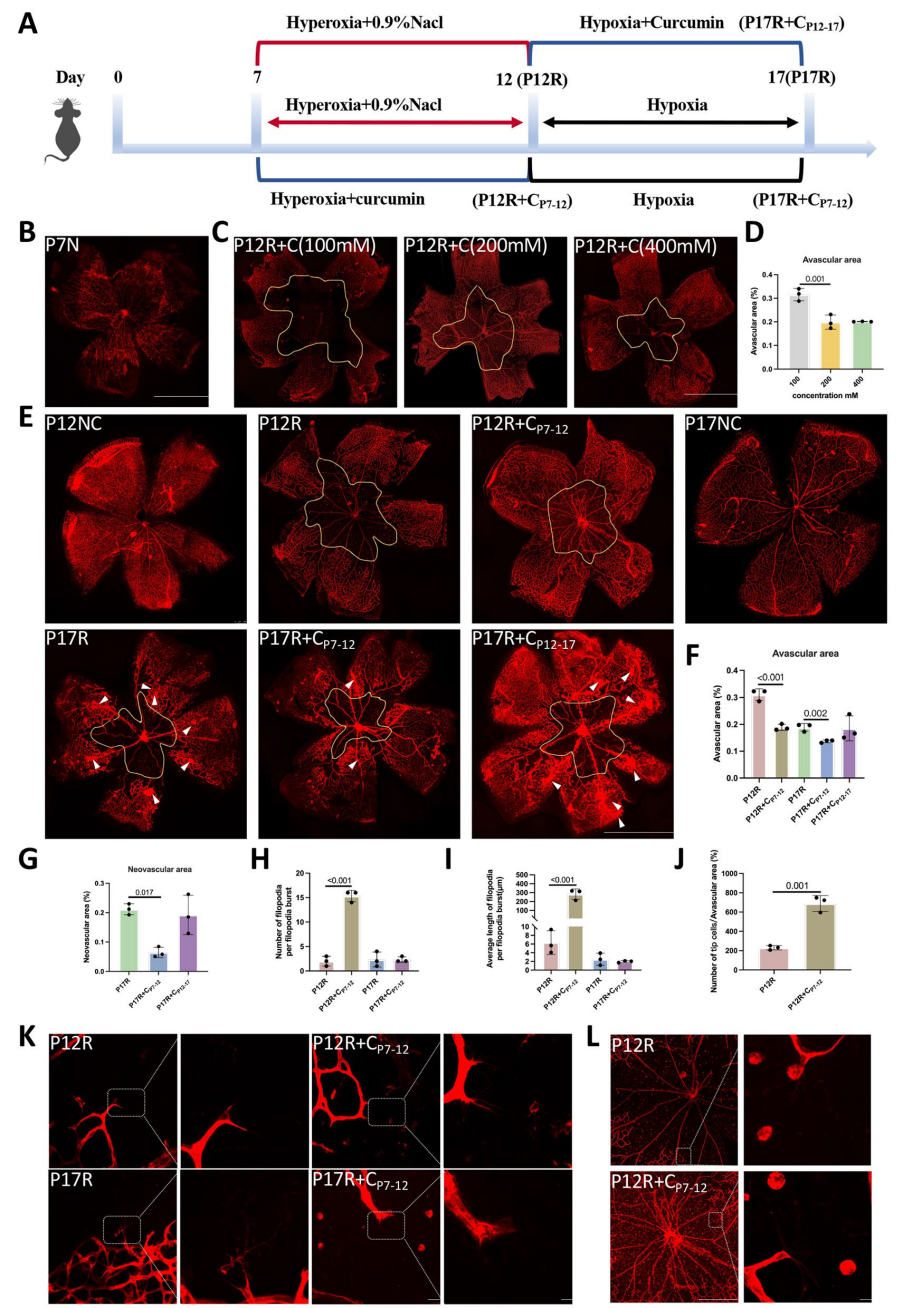
Curcumin decreases the avascular area in OIR mice under hyperoxia and enhances physiological blood vessel sprouting. **A** Mouse experiment flow diagram. **B** Using IB4 staining, the retinal blood vessels of P7 normal mice were detected in the retina (n = 3). **C** Retinal patches from OIR mice treated with 100 mM, 200 mM, or 400 mM curcumin at P12. (n = 3) (scale: 500 μm). **D** The percentage of avascular area/whole retina area in each group of mice. **E** Retinal patches from mice in the P12NC, P12R, P12R + C_P7-12_, P17NC, P17R, P17R + C_P7-12,_ and P17R + C_P12-17_ groups (n = 3) (scale bar: 500 μm). The newly proliferated blood vessels are indicated by white arrows. **F** Percentage of avascular area/whole retina in the P12R, P12R + C_P7-12_, P17R, P17R + C_P7-12_ and P17R + C_P12-17_ groups. **G** Percentage of neovascular area/whole retina in the aforementioned groups. **H**-**I** Quantitative statistics on the number and length of distal buds and filopodia. **J** Quantitative statistics of the number of tip cells in the avascular area. **K** Retinal patch analysis was used to assess vascular filopodia in the avascular area in the P12R, P12R + C_P7-12_, P17R and P17R + C_P7-12_ groups (n = 3) (scale bar: 25 μm). An enlarged image is shown in the white rectangle, with scale bars indicating 10 μm. **L** Tip cells in the avascular retinal region in the P12R and P12R + C_P7-12_ groups (n = 3) (scale bar: 10 μm)

A flow diagram of the mouse experiments is shown in Fig. 5A. On P7, the retinal blood vessels in the mice were not fully developed (Fig. 5B); therefore, high oxygen treatment could cause retinal vascular abnormalities. When the concentration of curcumin was 100 mM, there was no significant improvement in retinal blood vessels; when the concentration was 400 mM, the retinal vascular condition was close to that observed for mice treated with 200 mM; therefore, the drug concentration used for subsequent experiments was 200 mM (Fig. 5C, D).

In this study, we investigated the effect of curcumin on the development of retinal blood vessels in the OIR hyperoxia phase. Compared with those in the P12R group, the avascular area in the P12R + CP_7-12_ group was smaller, showing that curcumin decreased the avascular area and enhanced angiogenesis during the OIR hyperoxia phase (Fig. 5E, F). Compared with the P17NC group, the P17R group exhibited increased neovascularization. The avascular area and neovascularization area in the P17R + C_P7-12_ group was smaller than those in the P17R group, indicating that curcumin effectively protected blood vessels and reduced vascular occlusion during the hyperoxic period, thereby alleviating neovascular proliferation. However, the neovascularization area in the P17R + C_P12-17_ group did not decrease, indicating that curcumin does not have an inhibitory effect on neovascularization and only has a protective effect on retinal blood vessels during hyperoxia (Fig. 5E–G).

Tip cells of the vascular endothelium play an important role in vascular sprouting, and studying levels of vascular spreading (distal sprouts and filopodia) can help us better understand vascular proliferation. The P12R + C_P7-12_ group exhibited significantly greater numbers and lengths of distal sprouts and filopodia (Fig. 5H, I, K) as well as tip cells in the central avascular zone (Fig. 5J, L), indicating that curcumin protects vascular proliferation and alleviates vascular occlusion. However, compared with the P17R group, the P17R + C_P7-12_ group showed an improvement trend, but the difference was not significant (Fig. 5H, I, K). This indicates that curcumin has a significant protective effect on blood vessels during the high oxygen phase of OIR.

### During the high-oxygen phase of OIR, curcumin protects retinal astrocytes

**Fig. 6:**
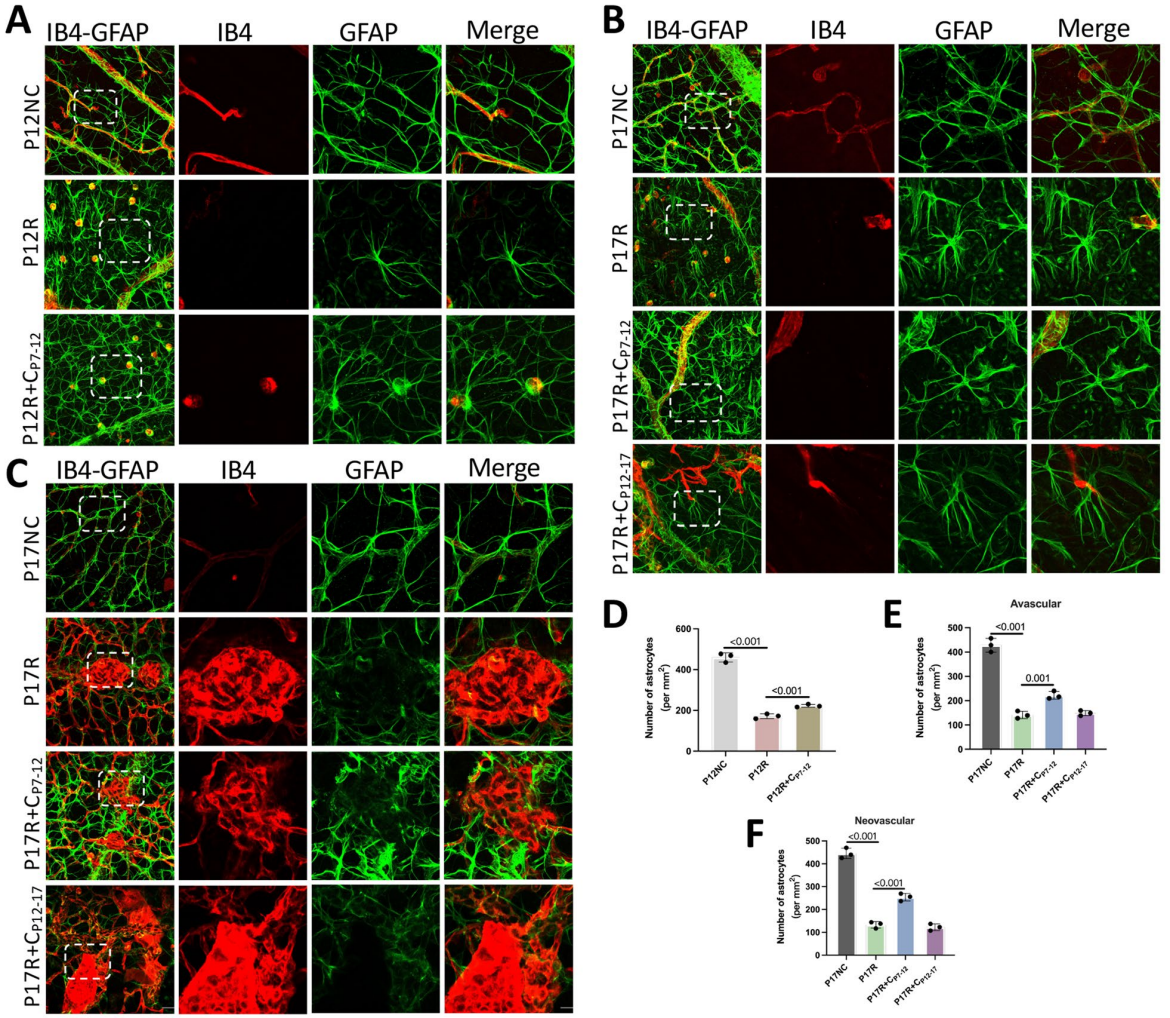
During the high oxygen phase of OIR, curcumin protects retinal astrocytes. **A** P12NC, P12R, and P12R + C_P7-12_ retinas were immunostained with GFAP (green) and IB4 (red). (Scale: 25 μm). An enlarged image is shown in the white rectangle, with scale bars indicating 10 μm. **B** The morphology and number of astrocytes in the avascular area of the retina of OIR mice in the P17NC, P17R, P17R + CP7-12, and P17R + CP12-17 groups were observed (n = 3). **C** The morphology and quantity of astrocytes in the neovascularized areas of each group were observed (n = 3). (Scale: 25 μm). An enlarged image is shown in the white rectangle, with scale bars indicating 10 μm. **D**–**F** The density of astrocytes in avascular or neovascularized areas of the retina of mice in each group was quantified

Blood vessels are constructed with astrocytes as the skeleton. The surviving retinal astrocytes in the P12NC and P17NC groups were immunolabeled for GFAP, and their dendritic shape and distribution were consistent (Fig. 6A–C). In comparison, the quantity of astrocytes in the avascular zone of the P12R group decreased dramatically, and their shape changed. However, compared with those in the P12R group, the morphology and quantity of astrocytes in the P12R + C_P7-12_ group normalized, indicating that curcumin can effectively ameliorate high oxygen-induced damage to astrocytes (Fig. 6A, D). Compared with those in the P17NC group, the number of astrocytes in the avascular and neovascularized areas in the P17R group was substantially lower, and the shape changed (Fig. 6B, C, E, F); however, the number and shape of astrocytes in the avascular and neovascularization regions in the P17R + CP_7-12_ group normalized, and there was no significant normalization in the P17R + CP_12-17_ group (Fig. 6B, C, E, F). This indicates that curcumin effectively protects blood vessels and reduces vascular occlusion during hyperoxia and that its ability to alleviate vascular damage is still effective in mice at 17 days. These findings also indicated that curcumin played a protective role in the hyperoxic period and effectively suppressed OIR progression and that curcumin administration during hypoxia had no significant effect.

### Retinal vascular occlusion is associated with ferroptosis

**Fig. 7:**
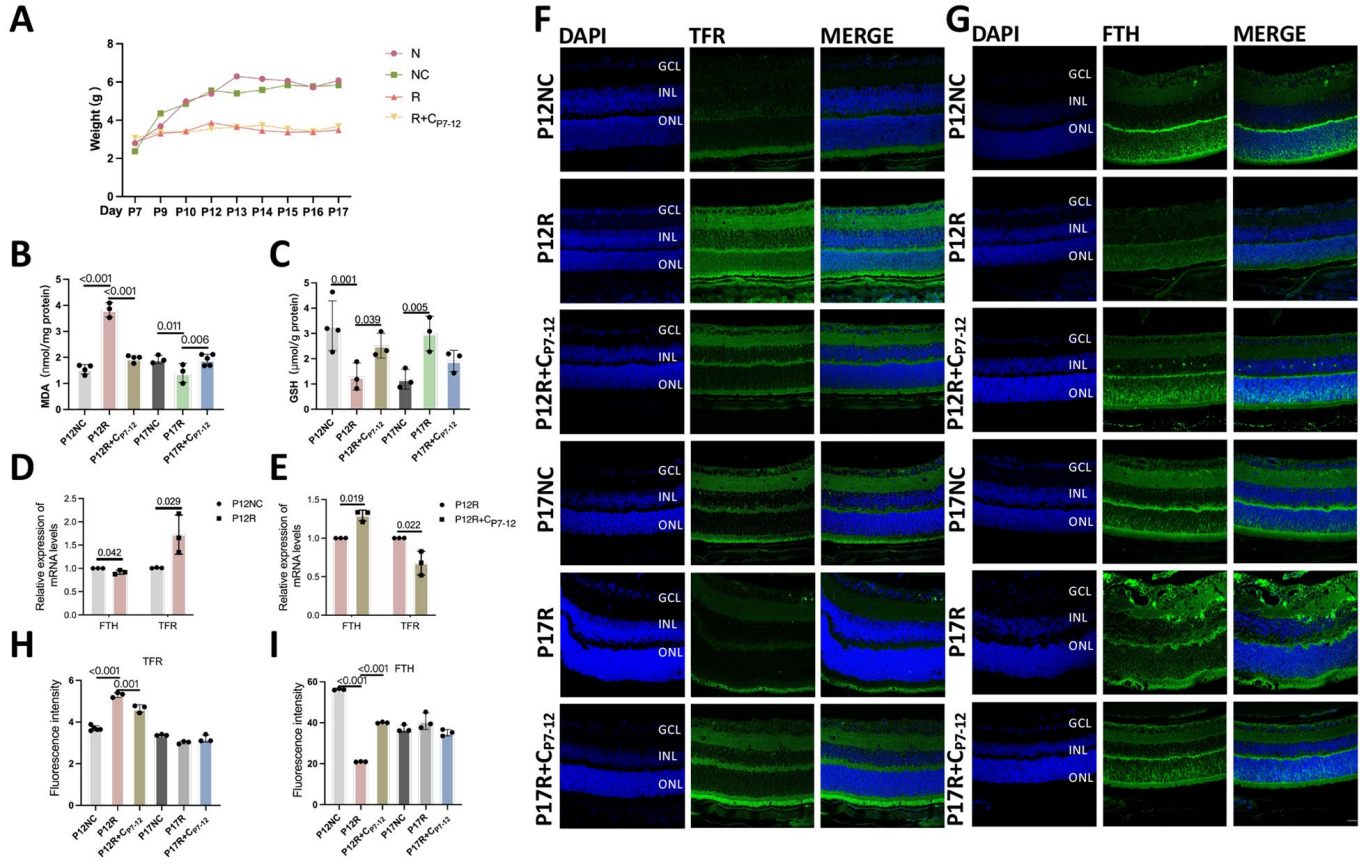
Retinal vascular occlusion is associated with ferroptosis. **A** Mouse weight changes are depicted schematically. **B**, **C** Changes in MDA and GSH levels in the retinas of mice in the P12NC, P12R, P12R + C_P7-12_, P17NC, P17R and P17R + C_P7-12_ groups (n ≥ 3). **D** The relative mRNA levels of FTH and TFR in the retinal membrane of mice in the NC and R groups (n = 3). **E** The relative mRNA levels of FTH and TFR in the retinal membrane of mice in the P12R and P12R + C_P7-12_ groups (n = 3). **F**, **G** TFR and FTH immunofluorescence intensity in mouse retinas (n = 3). The quantification of (**H**, **I**)

At different time intervals, there was no significant difference in body weight between the normal and control groups; there was no significant difference in body weight between the R and R + C_P7-12_ groups, indicating that curcumin did not interfere with mouse development (Fig. 7A). Compared to the P12NC group, the P12R group exhibited higher MDA expression, lower GSH expression (Fig. 7B, C), significantly higher TFR mRNA expression, and significantly lower ferritin heavy chain (FTH) mRNA expression (Fig. 7D). These findings indicate that ferroptosis intensifies in P12 OIR mice and that vascular occlusion is caused by ferroptosis. However, the MDA level significantly decreased in the P17R group, while the GSH level significantly increased, indicating decreased ferroptosis due to increased neovascularization during hypoxia (Fig. 7B, C). In the P12R + C_P7-12_ group, MDA was reduced, GSH was increased, TFR mRNA was reduced, and FTH mRNA was increased, indicating that curcumin can effectively inhibit ferroptosis (Fig. 7B, C, E). The fluorescence results were consistent with the PCR results (Fig. 7F–I). Curcumin suppresses ferroptosis during the hyperoxia phase of OIR.

### Ferroptosis is enhanced by curcumin via the CXCL10/CXCR3 axis

**Fig. 8:**
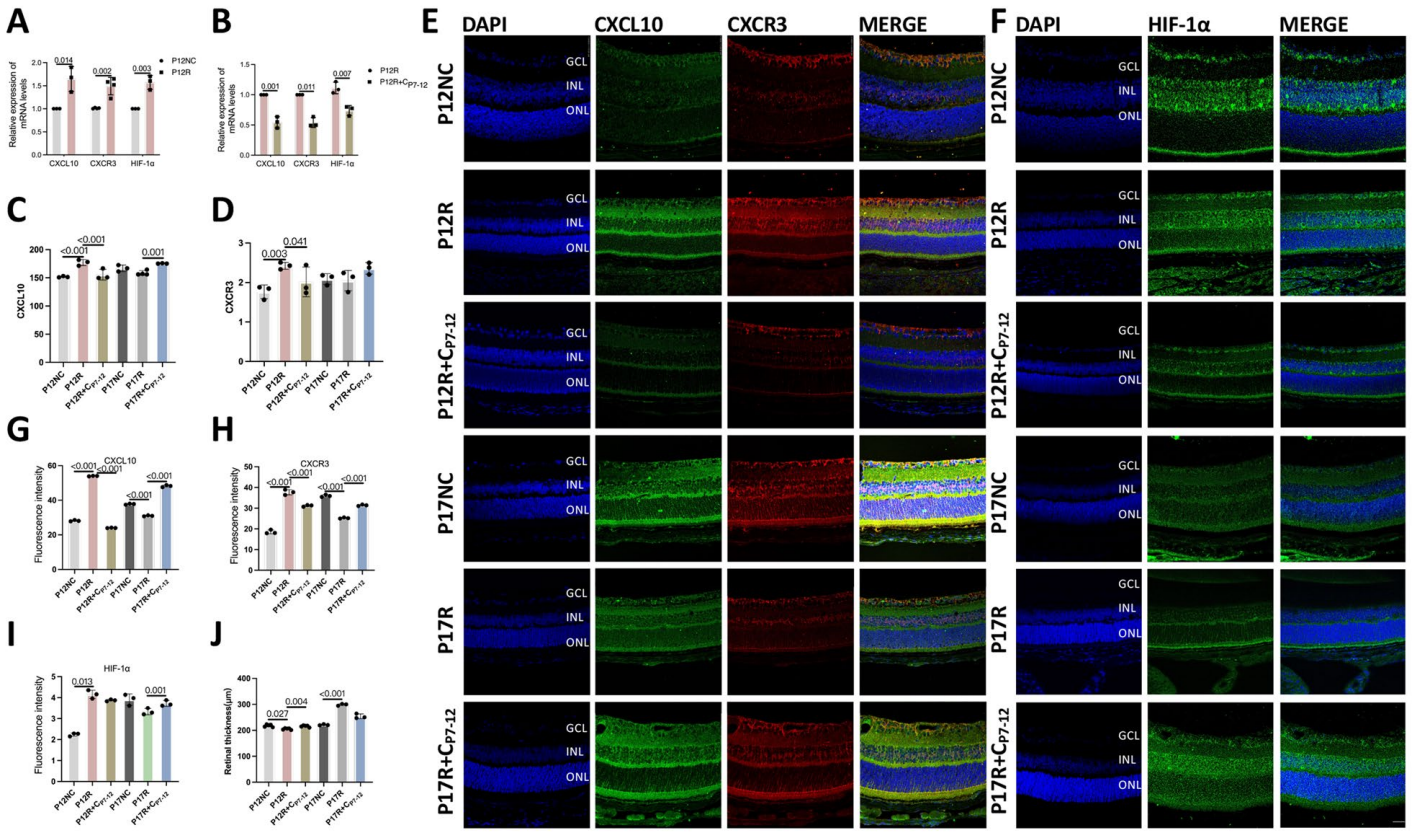
Curcumin inhibits ferroptosis via the CXCL10/CXCR3 axis. **A** Compared to those in the retinas of mice in the P12NC group, the mRNA expression levels of CXCL10, CXCR3 and HIF-1α in the retinas of mice in the P12R group were higher (n = 3). **B** Compared to those in the retinas of mice in the P12R group, the mRNA expression levels of CXCL10, CXCR3 and HIF-1α in the retinas of mice in the P12R + C_P7-12_ group were lower (n = 3). **C**, **D** CXCL10 and CXCR3 protein expression levels in the retinas of mice in each group (n ≥ 3). **E**, **F** CXCL10, CXCR3 and HIF-1α immunofluorescence intensity in mouse retinas (n = 3) (scale bar = 25 μm). The quantification of (**G**, **I**). **J** Statistics of retinal thickness in each group of mice

In contrast to the P12NC group, the P12R group showed increased mRNA expression of HIF-1α and mRNA and protein expression of CXCL10 and CXCR3; in contrast, the P12R + C_P7-12_ group showed decreased mRNA expression of HIF-1α and mRNA and protein expression of CXCL10 and CXCR3 (Fig. 8A–D). This indicates that during the high-oxygen period of OIR, HIF-1α and CXCL10/CXCR3 expression increases, and curcumin inhibits their expression. Compared to that in the P17N group, CXCL10/CXCR3 protein expression in the P17R group was lower but was not significantly different. We believe that due to the increased expression of CXCL10/CXCR3 in the retina during P7-P12 and the decreased expression of CXCL10/CXCR3 during P12-P17, there was no significant difference compared to those in the normal group (Fig. 8C, D). As a result, our primary research focus was on the ability of curcumin to protect the retina during the high oxygen phase of OIR. The fluorescence data confirmed this finding (Fig. 8E–I). The retinal thickness of the P12R group decreased as a result of retinal vessel obstruction, whereas the retinal thickness of the P17R group significantly increased as a result of increased neovascularization caused by ischemia and hypoxia. The retinal thickness increased in the presence of curcumin during the hyperoxia phase (Fig. 8J). Therefore, we believe that curcumin inhibits the expression of CXCL10/CXCR3.

### Factors downstream of CXCR3 related to ferroptosis

**Fig. 9:**
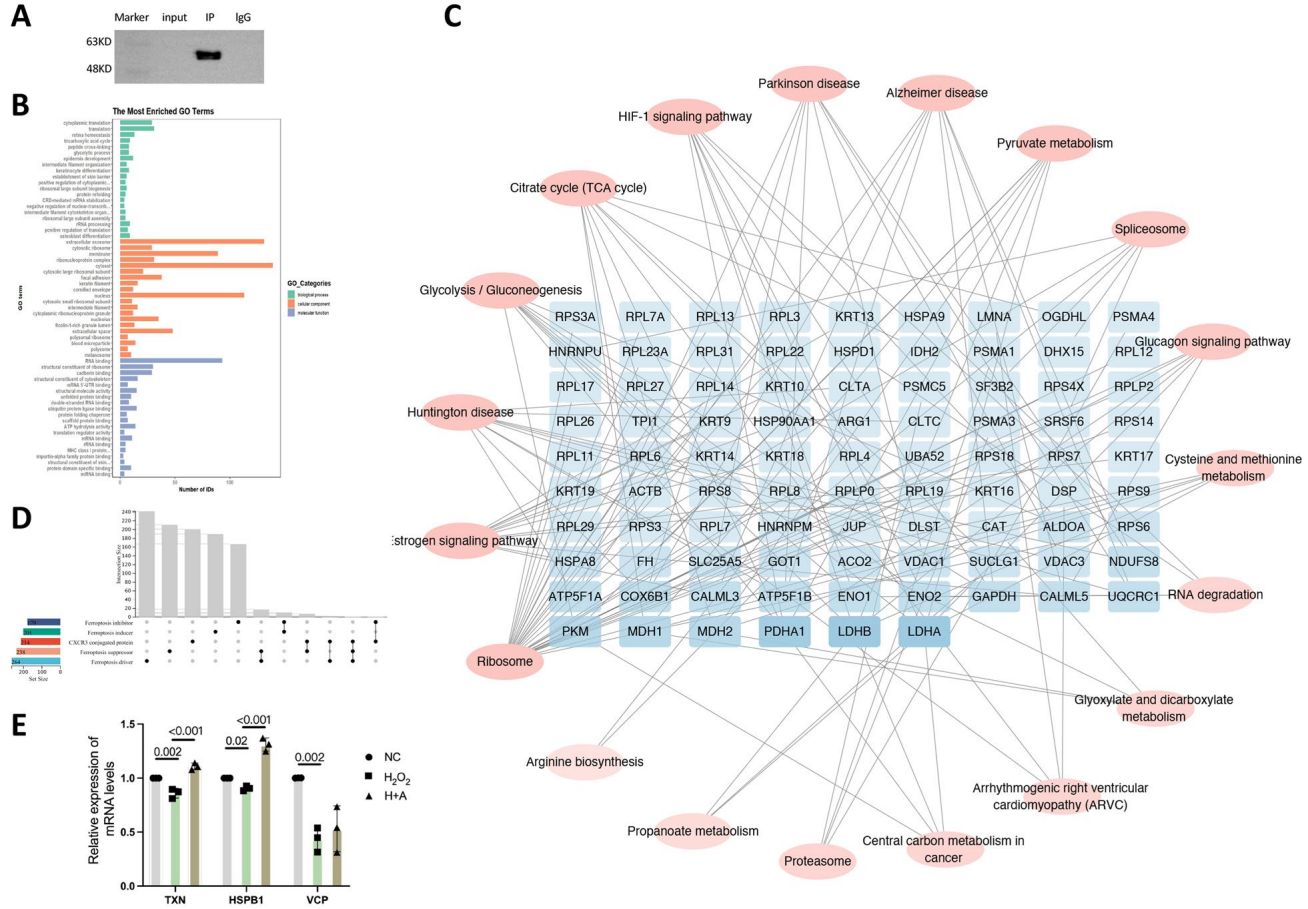
Factors downstream of CXCR3 related to ferroptosis. **A** CoIP of CXCR3 in normal HRMECs. **B** GO enrichment analysis of the CoIP results for CXCR3. **C** KEGG analysis of binding proteins. **D** Using the UpSet plot, overlapping proteins between binding proteins and ferroptosis suppressors, drivers, inducers and inhibitors were identified. **E** TXN, HSPB1, and VCP mRNA levels were compared among the NC, H_2_O_2_, and H + A groups

Next, we investigated the various mechanisms of ferroptosis mediated by CXCL10 and CXCR3. As an effector, CXCL10 interacts with the CXCR3 receptor and causes ferroptosis. We wondered whether CXCR3-related effector proteins are associated with ferroptosis. Protein‒protein interactions were investigated using CoIP (Fig. 9A). The protein concentration in the CoIP experiment was 1.59 ug/ul. CXCR3 can bind 214 proteins in HRMECs, and these proteins are involved in numerous pathways, such as ribosome-related pathways, estrogen signaling pathways, glycolysis/gluconeogenesis, citrate cycle (TCA cycle), and HIF-1 signaling pathways (Fig. 9B, C).

**Fig. 10:**
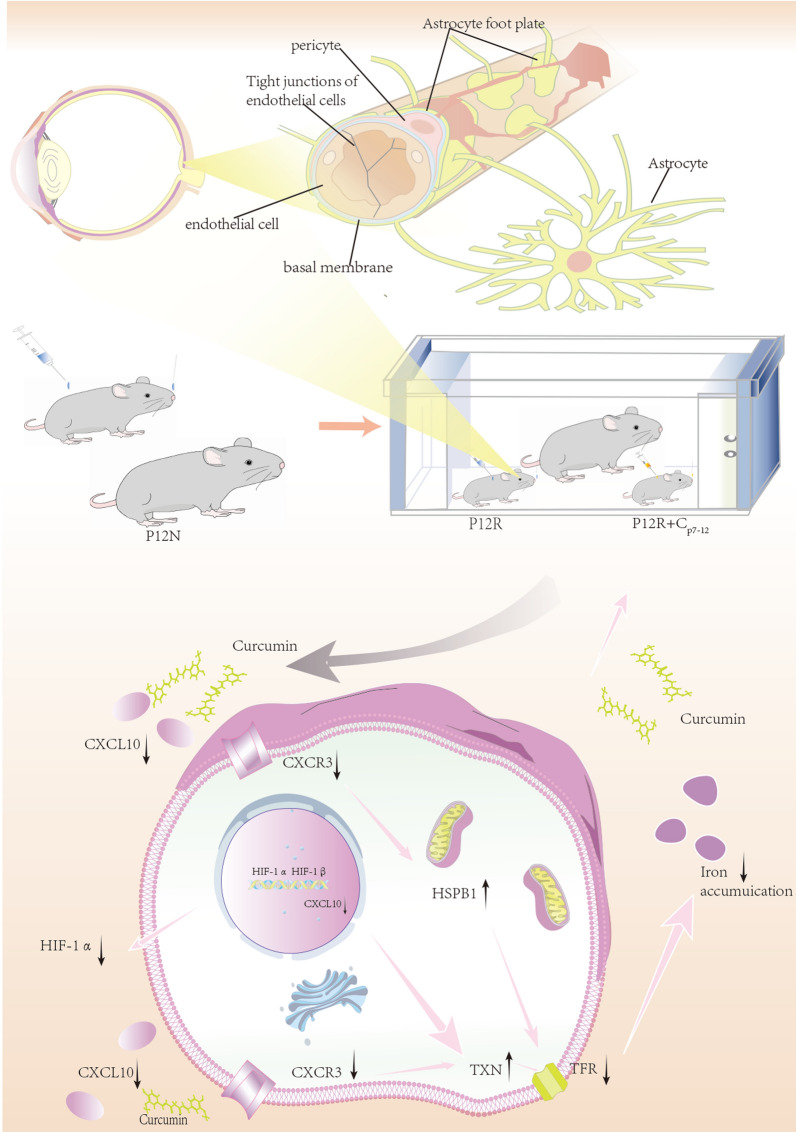
Schematic diagram of the mechanism

Fumarate hydratase (FH), phosphatase 1, regulatory (inhibitor) subunit 13 (PPP1R13L), Fatty acid binding protein 4 (FABP4), Peroxiredoxin-1 (PRDX1), thioredoxin-1 (TXN), Valosin containing protein (VCP), heat shock protein beta-1 (HSPB1), Isocitrate Dehydrogenase 2 (IDH2), Ribosomal Protein L8 (RPL8), ELAV Like RNA Binding Protein 1 (ELAVL1), Arachidonate 12-Lipoxygenase, 12R Type (ALOX12B), Glutamic-Oxaloacetic Transaminase (GOT1), and Catalase (CAT) are proteins that interact with ferroptosis drivers, inducers, inhibitors, and suppressors (Fig. 9D). Therefore, we believe that CXCR3 contributes to ferroptosis by binding to these proteins. Subsequently, N, H_2_O_2_, and H + A groups were constructed to verify the relevant factors involved in the exacerbation of ferroptosis by CXCL10/CXCR3 after oxidative stress in cells. The protein expression of TXN and HSPB1 varied with changes in CXCL10/CXCR3 in the N, H_2_O_2_, and H + A groups (Fig. 9E). Therefore, we concluded that CXCL10/CXCR3 aggravates ferroptosis in the retina through TXN and HSPB1. Schematic diagram of the mechanism by which curcumin reduces ferroptosis in the hyperoxia phase of OIR through the CXCL10/CXCR3 axis (Fig. 10).

## Discussion

ROP is the major cause of blindness in children, with hypoxia-induced neovascularization leading to subsequent retinal detachment and lifelong vision loss (Rivera et al. 2017; Fevereiro-Martins et al. 2022). Early clinical treatment focused mostly on stopping the formation of peripheral retinal blood vessels toward the serrated edge, and earlier research has also investigated medicines that impede neovascularization (Hartnett 2020). The hyperoxia phase is essential for the pathogenesis of ROP, and there is a link between the degree of hypoxia and the extent of aberrant neovascularization (Li et al. 2021; Shi et al. 2015; Miyamoto et al. 2002; Lan et al. 2021). If retinal blood vessels can develop regularly under hyperoxia, pathological angiogenesis caused by retinal hypoxia and ischemia can be avoided (Lan et al. 2021). However, the molecular processes underlying the evolution of ROP during hyperoxia still need to be clarified. We believe that during the hyperoxia phase of ROP, the expression and binding of CXCL10 and CXCR3 increase, ferroptosis of retinal endothelial cells intensifies, and the proliferation and migration of these cells decrease, resulting in vascular occlusion. Curcumin suppresses ROP progression by competitively binding to CXCL10, reducing CXCL10 binding to CXCR3, and reducing ferroptosis.

We examined blood samples from normal controls and ROP patients from the GEO database to identify major contributing factors, and CXCL10 was shown to be considerably increased in ROP patients. CXCL10 is an important chemokine that attracts monocytes, macrophages, microglia, and T lymphocytes (Zhu et al. 2017). Endothelial cells can secrete CXCL10, contributing to the progression of disorders such as retinal ischemia‒reperfusion, Alzheimer's disease, multiple sclerosis, and neuronal injury (Tokunaga et al. 2018); it binds to the CXCR3 receptor and can regulate the immune response by activating and attracting leukocytes (Lee et al. 2009).

In this study, we applied KEGG enrichment analysis to identify all the differential factors, and one significantly enriched pathway was ferroptosis. Previous studies have shown that ferroptosis induces CXCL10 expression, interfering with the iron metabolism pathway to replenish chemokines, leading to an increase in intracellular iron accumulation. In recent years, ferroptosis has been shown to be associated with multiple pathological diseases. Ferroptosis is characterized by ROS production and lipid peroxidation and has been found to be involved in retinal damage, such as photoreceptor degeneration and ROP exacerbation (Liu et al. 2023a). The accumulation of intracellular iron plays a role by promoting the binding of CXCL10 to its specific receptor CXCR3, which attracts infiltrating lymphocytes and activates microglia (Imaizumi et al. 2015). This is consistent with our results. Our study revealed that in an H_2_O_2_ cell model, oxidative stress increased the expression of CXCL10 and CXCR3, and when ferroptosis was inhibited or induced, both CXCL10 and CXCR3 varied with changes in ferroptosis. When the binding of CXCL10 to CXCR3 was blocked, ferroptosis was significantly reduced. Therefore, we believe that CXCL10 exacerbates ferroptosis through CXCR3 and that intracellular iron accumulation promotes the expression of CXCL10 and CXCR3.

Correlation analysis of ferroptosis-related factors with CXCL10 revealed that the TFR, HIF-1α, STAT3 and PTGS2 were significantly correlated with CXCL10. Subsequently, an H_2_O_2_ cell model was constructed for validation. After 9 h of stimulation, the expression of CXCL10, CXCR3, TFR, STAT3, and PTGS2 increased, and HIF-1α expression decreased. Then, the expression of related factors was observed by constructing cell models using ferroptosis inhibitors and inducers. When ferroptosis was induced or inhibited, the expression of CXCL10, CXCR3, TFR, STAT3 and PTGS2 changed accordingly. Therefore, we concluded that TFR, STAT3 and PTGS2 aggravate ferroptosis after interacting with CXCL10/CXCR3 and that HIF-1α inhibited ferroptosis. This finding is consistent with previous conclusions. HIF-1 is known as a housekeeping gene because most hypoxia-induced signals are generated through HIF-1. HIF-1α is an active subunit of HIF-1 that rapidly degrades under normoxic conditions; however, hypoxia blocks this degradation (Li et al. 2023). Accumulated HIF-1α binds to hypoxia inducible factor 1B to form the HIF-1 complex, which then promotes the binding of hypoxia response elements (HREs) to the solute carrier family 2-member 12 (SLC2A12) promoter through HIF-1/HRE signaling, thereby exerting an inhibitory effect on ferroptosis (Li et al. 2023). However, when ferroptosis is activated, HIF-1 negative feedback is exacerbated due to fluctuations in HIF-1 expression. Considering the consistency among animal experiment results, relevant studies have chosen to construct models via 12-h stimulation. STAT3 is a key oncogene with dual functions of signal transduction and transcriptional activation. STAT3 overactivation is a key event in the formation of most human cancers and plays a crucial role in cell proliferation, angiogenesis, metastasis, and immune suppression (Ouyang et al. 2022). CXCL10 inhibits SLC7A11 by binding to CXCR3 and promotes the phosphorylation of STAT3. In addition, inhibiting STAT3 can reverse ferroptosis exacerbated by CXCL10. In addition, the activation of STAT3/SLC7A11 signaling is also eliminated by the inhibition of CXCR3 (Liang et al. 2023; Zhang et al. 2022b). PTGS2, also known as cyclooxygenase 2, is a marker gene for ferroptosis, and its activation can exacerbate ferroptosis (Li et al. 2022). Therefore, we speculate that during the high-oxygen phase of ROP, HIF-1α, STAT3 and PTGS2 are associated with ferroptosis exacerbated by the CXCL10/CXCR3 axis.

Curcumin is an inhibitor of CXCL10. Curcumin is a natural antioxidant with anticancer, antiviral, antiarthritic, antioxidant, anti-inflammatory and immunomodulatory properties (Yang et al. 2021). By triggering NRF2 to inhibit CXCL10 expression and reverse ferroptosis, NRF2 has a protective effect on endothelial cells (Yuan et al. 2017; Ashrafizadeh et al. 2020; Zhou et al. 2023). Subsequently, we searched for targets associated with ROP and curcumin. We discovered 38 overlapping targets, one of which was CXCL10. PTGS2 and STAT3 were identified as factors associated with ferroptosis, a finding that is consistent with our previous research. In addition, curcumin binds to CXCL10/CXCR3. Therefore, we hypothesized that curcumin can inhibit ROP progression by blocking ferroptosis through the CXCL10/CXCR3 axis.

Following the addition of curcumin to H_2_O_2_-treated cells, the CXCL10 and CXCR3 mRNA and protein levels decreased, and changes in the TFR mRNA, MDA, and GSH levels suggested that curcumin suppressed CXCL10/CXCR3 signaling and ferroptosis. Cell proliferation and migration also increased or decreased in response to blocking or enhancing ferroptosis, suggesting that ferroptosis can impact retinal vascular endothelial cells and, consequently, retinal vascular architecture. The reduction in cell migration and proliferation induced by enhanced ferroptosis could be reversed by curcumin. Curcumin may be able to prevent high oxygen-induced vascular occlusion and safeguard retinal endothelial cell function. Thus, to investigate the impact of curcumin on retinal blood vessels in OIR, we generated an OIR mouse model.

This is the first study to investigate the influence of curcumin on the formation of retinal blood vessels. Curcumin reduced the avascular area and increased neovascularization in the hyperoxia phase of OIR, demonstrating that curcumin has a protective effect on retinal blood vessels and can inhibit the damage caused by high oxygen levels in blood vessels. However, curcumin alone did not reduce the avascular or neovascularized area in the hypoxia phase or OIR, showing that curcumin plays a role in the hyperoxia phase of OIR. Curcumin can protect arteries from hyperoxia damage by protecting distal sprouts and filopodia, increasing the number of tip cells, and decreasing vascular occlusion in the hyperoxia phase of OIR. During normal angiogenesis, endothelial cells are produced, and tip cells play an important role in vascular sprouting (Eelen et al. 2015; Pitulescu et al. 2017). The foundation of blood vessel development is provided by astrocytes. The number and length of distal sprouts and filopodia considerably increased after treatment with curcumin in the hyperoxia group, and the shape and quantity of astrocytes returned to normal levels. Therefore, curcumin alleviates vascular blockage induced by high oxygen levels through multiple mechanisms, such as endothelial cell sprouting and the formation of an astrocyte cytoskeleton, which is good for regular blood vessel growth. The mechanism by which curcumin protects retinal blood vessels in OIR mice and inhibits OIR in vitro have been validated in animal experiments.

Ferroptosis is a major cause of retinal vascular ischemia and occlusion in OIR mice. Hyperoxia suppresses growth factors in preterm newborns with retinal hypoplasia, halting retinal vascular formation. Growth factors stimulate vascular proliferation under normoxic conditions, leading to ROP (Hellström et al. 2013). The mechanism by which high oxygen inhibits vascular growth is to trigger vascular endothelial cell ferroptosis. Ferroptosis is a novel type of cell death frequently accompanied by the substantial accumulation of lipid peroxides (Moos et al. 2022). Hypoxia activates ferroptosis during ischemia of the heart and brain blood arteries, which causes cell damage and diseases, including myocardial ischemia and stroke (Ma et al. 2022; Lv et al. 2021; Liu et al. 2023b). Although TFR expression increases and accelerates transport, excessive iron ions induce ferroptosis. According to the results of our study, retinal vascular blockage and ferroptosis are important factors in OIR-induced retinopathy in mice. In the P12R group, MDA and TFR mRNA expression increased dramatically, but GSH and FTH mRNA levels decreased. Immunofluorescence revealed that OIR hyperoxia-induced vascular occlusion was caused by ferroptosis. We believe that hyperoxia leads to the accumulation of iron ions in endothelial cells. However, P17 OIR mice were hypoxic, and when new blood vessels grew, ferroptosis decreased. Individual differences in ferroptosis existed due to the effect of vascular occlusion on changes in total MDA and GSH content.

Following the administration of curcumin, there was a decrease in ferroptosis and in HIF-1α and CXCL10/CXCR3 expression. This finding is in line with in vitro studies. Curcumin protects blood vessels during the high oxygen phase of OIR by suppressing the production of CXCL10/CXCR3, which in turn reduces ferroptosis in animal models. Furthermore, we analyzed the body weights of the mice at various time points and discovered that the curcumin injection did not increase or decrease the body weights of the OIR mice. Curcumin was shown to have no adverse effects on OIR mice and could be used as a medication to protect blood vessels.

Next, we examined the downstream factors of CXCR3. CXCL10 induces ferroptosis after interacting with the CXCR3 receptor. We wondered whether CXCR3-related effector proteins are associated with ferroptosis. CoIP-seq revealed that CXCR3 binds proteins associated with ribosomes, the estrogen signaling pathway, glycolysis/gluconeogenesis, the TCA cycle, and the HIF-1 signaling pathway, suggesting that CXCR3 is involved in cellular energy metabolism and is associated with mitochondrial function. Overlapping protein analysis and ferroptosis validation were subsequently performed for FH, PPP1R13L, FABP4, PRDX1, TXN, VCP, HSPB1, IDH2, RPL8, ELAVL1, ALOX12B, GOT1, and CAT. TXN, VCP, and HSPB1 expression was altered in the H_2_O_2_ group. Changes in CXCR3/CXCL10 affected TXN and HSPB1. TXN is a 12 kDa protein that reduces oxidative stress and inhibits ferroptosis (Bai et al. 2021; Lin et al. 2023). HSBP1 can inhibit elastin-induced ferroptosis in tumor cells (Wang et al. 2022b; Dai and Hu 2022). VCP is a widely expressed protein that participates in various cellular processes, such as cell cycle regulation, nuclear membrane development, Golgi biogenesis, and the ubiquitin‒proteasome system; it protects cells from stress by modifying mitochondrial function via alterations in mitochondrial localization (Ogor et al. 2021). We speculate that CXCR3 contributes to ferroptosis by binding to TXN and HSPB1, establishing the groundwork for understanding the mechanics of the CXCL10 and CXCR3 pathways and providing new therapeutic options for the hyperoxia phase of OIR.

In conclusion, CXCL10 is highly expressed in ROP patients. OIR mice exhibit vascular occlusion during the hyperoxia phase due to increased ferroptosis, and curcumin can reduce ferroptosis and restore vascular status via the CXCL10/CXCR3 axis. In conjunction with TXN, HSPB1, and other factors, CXCR3 may cause ferroptosis. Our experiment has several limitations, and pathway crosstalk is possible. CXCL10/CXCR3, for example, is involved in other processes, such as inflammation and oxidative stress.

## Supplementary Information

Supplementary Material 1.

## Data Availability

Data will be made available on request.
