# Supplementary material for: Curcumin inhibits ferroptosis-mediated vascular occlusion by regulating the CXCL10/CXCR3 axis in retinopathy of prematurity
Source: Mol Med. 2025 Mar 24;31:113. doi: 10.1186/s10020-025-01161-1 (PMC11934774; doi:10.1186/s10020-025-01161-1)
Supplement: Supplementary file 1 — Supplementary Material 1. [file 10020_2025_1161_MOESM1_ESM.docx]

Supplementary material


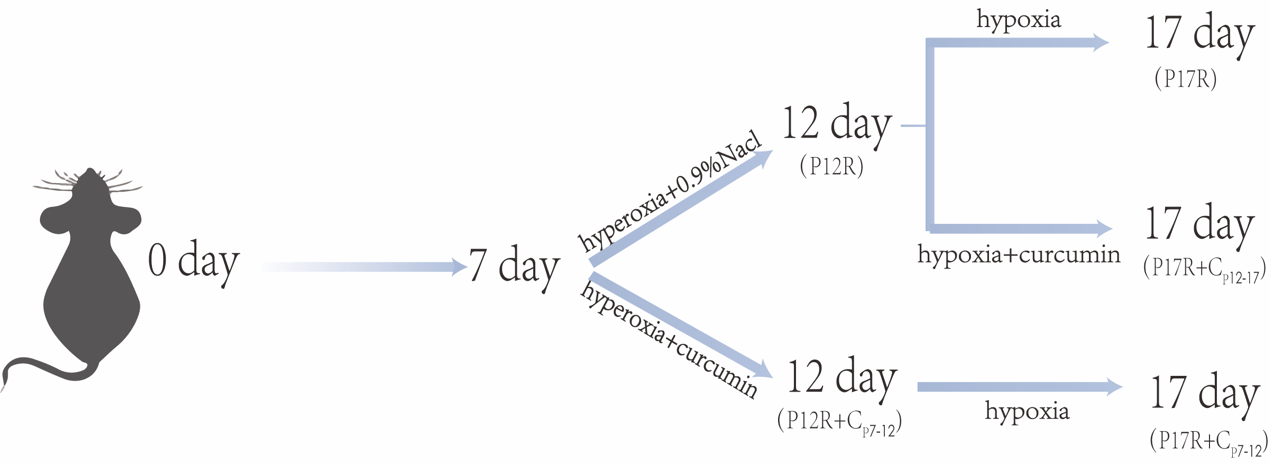


**Fig. Supplementary 1. Mouse experiment flow diagram.**


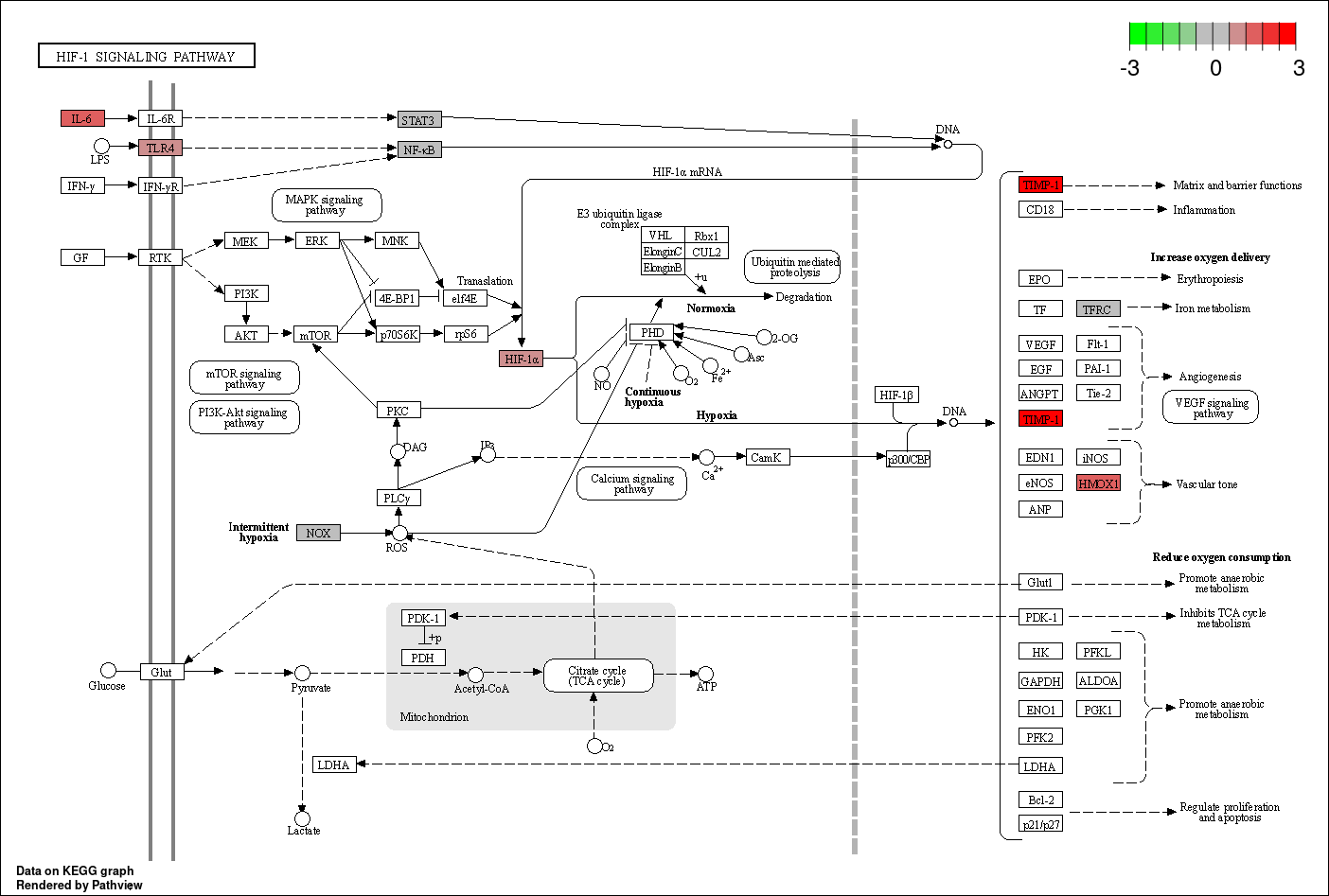


**Fig. Supplementary 2. HIF-1 pathway diagram of KEGG enrichment.** KEGG enrichment analysis was performed on differentially expressed factors that overlap with ferroptosis in GSE51039.we found that TFRC is a downstream factor in the HIF-1 pathway. The HIF-1 pathway may affect the simultaneous action of multiple factors. Color represents Fold Change.
